# Hybrid Outer Membrane Vesicles with Genetically Engineering for Treatment of Implant‐Associated Infections and Relapse Prevention Through Host Immunomodulation

**DOI:** 10.1002/advs.202415379

**Published:** 2025-02-14

**Authors:** Zhichao Wang, Mingfei Li, Wenshuai Li, Liuliang He, Long Wang, Kehan Cai, Xiao Zhao, Yazhou Chen, Daifeng Li

**Affiliations:** ^1^ Department of Orthopedics The First Affiliated Hospital of Zhengzhou University Zhengzhou 450052 China; ^2^ Medical 3D Printing Center The First Affiliated Hospital of Zhengzhou University Henan Institute of Advanced Technology of Zhengzhou University Zhengzhou 450052 China; ^3^ CAS Key Laboratory for Biomedical Effects of Nanomaterials and Nanosafety CAS Center for Excellence in Nanoscience National Center for Nanoscience and Technology of China Beijing 100190 China

**Keywords:** bacterial outer membrane vesicles, bone marrow targeting, immunomodulation, implant‐associated infections, relapse prevention

## Abstract

Implant‐associated infections (IAIs) are refractory to elimination, and the local immunosuppressive microenvironment (IME) exacerbates therapeutic difficulties, ultimately causing persistence and relapse. Therefore, exploring immunostrengthening treatments holds great promise for reversing IME and thoroughly eradicating chronic or repetitive infections. Bacterial outer membrane vesicles (OMVs) have emerged as potential immunostimulatory candidates; however, they lack active targeting capabilities and cause non‐specific inflammatory side effects. In this study, bone marrow‐derived mesenchymal stem cells (BMSCs) are genetically engineered to overexpress CXCR4 and isolated cell membranes (mBMSC_CXCR4_) for hybridization with OMVs derived from *Escherichia coli* (*E. coli*) to produce nanovesicles (mBMSC_CXCR4_@OMV). The resulting mBMSC_CXCR4_@OMV nanovesicles demonstrate excellent bone marrow targeting capability and are effectively taken up by bone marrow‐derived macrophages, triggering the efficient transition to pro‐inflammatory M1 status through TLR/NF‐κB pathway. This alteration promotes innate bactericidal capacity and antigen presentation. Subsequent activation of T and B cells and inhibition of myeloid‐derived suppressor cells (MDSCs) facilitated in vivo adaptive immunity in mouse models. Additionally, mBMSC_CXCR4_@OMV boosted memory B cell and bacteria‐specific antibody responses. Together, these data highlight the potential of mBMSC_CXCR4_@OMV to eradicate complicated IAIs and provide whole‐stage protection against postsurgical relapse, thus marking a significant immunotherapeutic advancement in the post‐antibiotic era.

## Graphical abstract

An immune‐strengthening nanovesicle, mBMSC_CXCR4_@OMV, is constructed by the hybridization of *Escherichia coli‐*derived outer membrane vesicles and mBMSC_CXCR4_ isolated from genetically‐engineered bone marrow‐derived mesenchymal stem cells overexpressing CXCR4. The nanovesicle mBMSC_CXCR4_@OMV selectively accumulates in the infected bone sites, and initiates both innate and adaptive immunity, thereby eradicating complicated implant‐associated infections and providing whole‐stage protection against postsurgical relapse.

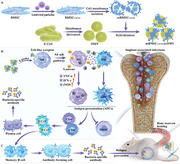

## 1 Introduction

Implant‐associated infections (IAIs), a disastrous complication in orthopedics, represent a major clinical challenge.^[^ ^1^, ^2^ ^]^ In particular, IAIs trigger bone destruction, compromise implant longevity, and require multiple subsequent surgeries, thus posing a threat to the life of the patient.^[^ ^3^, ^4^, ^5^ ^]^ Clinically, large doses of antibiotics are required to treat IAIs. However, these bone‐related infections exhibit low responsiveness to antibiotics due to the unique physiological bone structures.^[^ ^6^, ^7^ ^]^ Long‐term overdose also increases bacterial resistance and causes serious adverse reactions.^[^ ^8^, ^9^ ^]^ To tackle the crisis of antibiotic resistance, immunotherapy, as an antibiotic‐free strategy, is expected to eradicate infections by modulating the host immune system. For IAIs, the immunosuppressive microenvironment exacerbates therapeutic difficulty and causes the persistence and recurrence of such infections.^[^ ^10^, ^11^, ^12^ ^]^ The local hypoxic, nutrient‐deprived, and acidic infectious microenvironment in the deep layer of the bone can reprogram host immune cells toward anti‐inflammatory phenotypes.^[^ ^13^, ^14^ ^]^ Additionally, invading pathogens can camouflage their antigen epitopes to escape immune recognition and antigen presentation, and even trigger the death of antigen‐presenting cells (APCs) and lymphocytes.^[^ ^15^, ^16^, ^17^ ^]^ Therefore, immune‐strengthening therapeutics hold great promise for reversing suppressive host immunity, eradicating infections, and preventing relapses.

Bacterial outer membrane vesicles (OMVs), which are secreted by gram‐negative bacteria, possess a structure and composition similar to that of the bacterial outer membrane.^[^ ^18^, ^19^ ^]^ Due to the presence of abundant pathogen‐associated molecular patterns (PAMPs) on their surface, OMVs have been demonstrated to engage the immune system (including macrophages and dendritic cells [DCs]) and achieve therapeutic effects that have been preclinically investigated in several diseases such as cancer and infection.^[^ ^8^, ^20^, ^21^, ^22^, ^23^ ^]^ Additionally, OMVs can be produced in large quantities via straightforward bacterial fermentation and purification.^[^ ^22^, ^23^, ^24^ ^]^ Taken together, these properties identify OMVs as immunostimulatory candidates to reverse the immunosuppressive microenvironment of bacterial infections. However, OMVs lack active targeting capabilities, making them prone to rapid recognition and clearance by the immune system when administered intravenously.^[^ ^25^, ^26^, ^27^ ^]^ Furthermore, antigens on their surfaces can induce systemic inflammatory storms that hinder their potential for further in vivo applications.^[^ ^25^, ^26^, ^27^ ^]^

Bone marrow is a pivotal lymphoid organ that significantly impacts the regulation of immune responses.^[^ ^28^, ^29^, ^30^ ^]^ It supports the differentiation of hematopoietic stem cells (HSCs) into lymphocytes, and supervises the maturation and migration of immune cells, including macrophages, DCs, T cells, and B cells that collectively contribute to the modulation and enhancement of innate and adaptive immunity.^[^ ^29^, ^30^, ^31^ ^]^ Generally, invading microbials adhere to the surface of the intraosseous implants and further destroy the local bone marrow cavity during the progression of IAIs.^[^ ^1^, ^2^, ^3^, ^4^, ^5^ ^]^ Therefore, the targeted delivery of OMVs to the bone marrow potentially activates regional immunity to control infections, avoiding excessive activation of systemic immune organs. However, accurate targeting of bone marrow still remains challenging. Bone marrow‐derived mesenchymal stem cells (BMSCs) inherently exhibit the capacity to migrate toward both the bone marrow and sites of injury, while nanomaterials coated with their membranes have been shown to enhance homing capabilities to the bone marrow.^[^ ^32^, ^33^, ^34^ ^]^ Recently, several studies have revealed that stromal cell‐derived factor‐1 (SDF‐1) is often elevated at injury sites and acts as a strong chemoattractant that recruits circulating or resident BMSCs through its interaction with the C‐X‐C chemokine receptor 4 (CXCR4) receptor on the cell surface.^[^ ^35^, ^36^ ^]^ Furthermore, researchers suggest that during inflammatory responses, the cellular products enhance the interaction between SDF‐1 and CXCR4, and thereby augment the chemotactic migration of BMSCs to the injury site.^[^ ^37^, ^38^, ^39^ ^]^ However, CXCR4 is present in only a small proportion of BMSCs and its expression tends to diminish as the cells are expanded in vitro.^[^ ^33^, ^34^ ^]^ This reduction in CXCR4 expression limits the effectiveness of BMSCs in vivo. Given the crucial role of the CXCR4‐SDF‐1 axis in guiding cell movement, maintaining high levels of CXCR4 in BMSCs is essential for targeting damaged bone marrow sites.

**Scheme 1:**
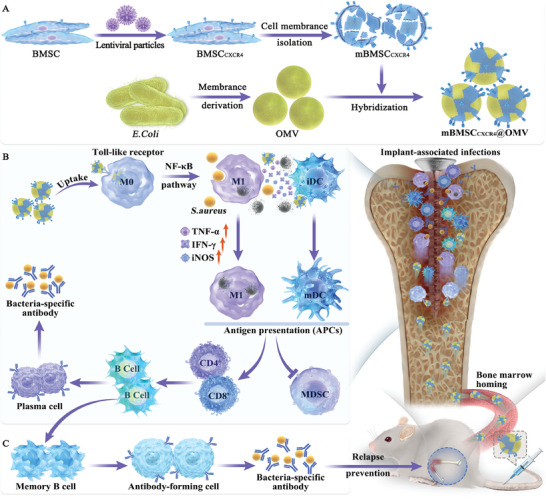
Illustration of the construction of mBMSC_CXCR4_@OMV nanovesicles and their immunotherapeutic effect to eradicate complicated IAIs and prevent relapse.

Hence, we genetically engineered BMSCs to overexpress CXCR4 using lentiviral particles and isolated their cell membranes (mBMSC_CXCR4_). Subsequently, we hybridized mBMSC_CXCR4_ with *Escherichia coli* (*E. coli*)‐derived OMVs to finally produce mBMSC_CXCR4_@OMV nanovesicles. This combination of dual‐functional membrane structures integrates their unique properties, potentially enhancing the efficacy of bone marrow‐targeting delivery of immunostimulatory OMVs. Our data demonstrated an obvious accumulation of mBMSC_CXCR4_@OMV in the infected bone area, and the nanovesicles were prone to uptake by bone marrow‐derived macrophages (BMDM). Further in vitro experiments revealed that mBMSC_CXCR4_@OMV efficiently switched M0 macrophages to M1 polarization via the activation of TLR/NF‐κB pathway, exhibiting superior phagocytosis and bactericidal capacity in vitro. This alteration also promoted M1 macrophages to present bacterial antigens by modulating the major histocompatibility complex II (MHC‐II) and immune co‐stimulatory molecules. Subsequent activation of T and B cells derived from the bone marrow and inhibition of myeloid‐derived suppressor cells (MDSCs) facilitated the in vivo clearance of infections through adaptive immunity in implant‐associated femoral osteomyelitis mouse models. Additionally, mBMSC_CXCR4_@OMV boosted memory B cells and bacteria‐specific antibody responses, thereby providing whole‐stage protection to prevent post‐surgical infection relapse (Scheme **1**). Therefore, this study opens up the possibility of using bone marrow‐targeted immunotherapy to eradicate complicated IAIs and prevent relapse, thus marking a significant immunotherapeutic advancement in the post‐antibiotic era.

## 2 Results and Discussion

### 2.1 Preparation and Characterization of mBMSC_CXCR4_@OMV

**Figure 1:**
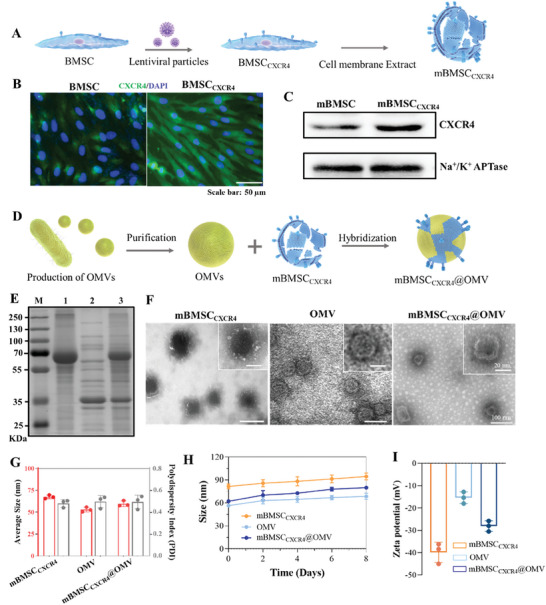
Fabrication and characterization of mBMSC_CXCR4_@OMV. A) Schematic diagram of mBMSC_CXCR4_ preparation procedures. B) Fluorescence microscope images of CXCR4 in mBMSC before (left) and after (right) gene transfection. Blue: DAPI; Green: CXCR4. C) Western blot analysis of CXCR4 and Na^+^/K^+^ APTase in mBMSC and mBMSC_CXCR4_. D) Schematic diagram of expression and purification of OMV. E) SDS‐PAGE analysis of mBMSC_CXCR4_, OMV, and mBMSC_CXCR4_@OMV. F) TEM images of mBMSC_CXCR4_, OMV, and mBMSC_CXCR4_@OMV. Scale bars: 20, and 100 µm, respectively. G) Hydrodynamic size distribution and PDI of mBMSC_CXCR4_, OMV, and mBMSC_CXCR4_@OMV. H) Stability of mBMSC_CXCR4_, OMV, and mBMSC_CXCR4_@OMV in PBS at 4 °C. I) Zeta potential of mBMSC_CXCR4_, OMV, and mBMSC_CXCR4_@OMV. All values are expressed as the mean ± SD, with *n* = 3 independent samples.

CXCR4 levels in BMSCs are essential for targeting damaged bone marrow sites.^[^ ^40^, ^41^ ^]^ First, we engineered BMSCs to enhance CXCR4 expression on the cell membrane using lentiviral particles that were generated by transfecting HEK 293T cells with the lentiviral expression plasmid pLV3‐EF1a‐CXCR4‐Puro along with the packaging plasmids pVSVg and psPAX2 (Table S1, Supporting Information). Subsequently, we extracted the cell membrane to obtain mBMSC_CXCR4_ (**Figure** **1**A) and further validated the overexpression of CXCR4 in this membrane structure. Fluorescence imaging revealed robust CXCR4 expression on BMSC membranes, as evidenced by enhanced fluorescence (Figure 1B). Western blot analysis also indicated a significant increase in CXCR4 protein levels compared to those in the control samples (Figure 1C; Figure S1, Supporting Information). These results suggested the successful genetic modification and expression of CXCR4 on BMSC membranes, laying the groundwork for further investigation of the functionality and applications of mBMSC_CXCR4_. Subsequently, OMVs were isolated from *E. coli* by high‐speed centrifugation as previously described.^[^ ^20^, ^21^ ^]^ The generated *E. coli*‐derived OMVs were fused to mBMSC_CXCR4_ using cell membrane hybridization, and this facilitated the incorporation of mBMSC_CXCR4_ into the OMV membrane (Figure 1D). SDS‐PAGE analysis confirmed that these proteins from both engineered mBMSC_CXCR4_ and OMV were present in the mBMSC_CXCR4_@OMV complex (Figure 1E). Transmission electron microscopy (TEM) images indicated that mBMSC_CXCR4_, OMV, and mBMSC_CXCR4_@OMV all exhibited a spherical morphology (Figure 1F), and their average diameters, as revealed by dynamic light scattering (DLS), were 67.5, 52.9, and 59.7 nm, respectively (Figure 1G). Moreover, to demonstrate that mBMSC_CXCR4_@OMV was not merely a mixture but also a true conjugate of mBMSC_CXCR4_ and OMV, a co‐localization experiment was conducted. The observed overlap of green and red fluorescence in the same area indicated the successful construction of mBMSC_CXCR4_@OMV (Figure S2, Supporting Information) compared to the separate fluorescence signals from mBMSC_CXCR4_ plus OMV. For further verification, we conjugated the His‐tag antibody and CXCR4 antibody with 5 and 10 nm gold nanoparticles, respectively, and then incubated them with mBMSC_CXCR4_@OMV. The TEM observation showed that the surface of mBMSC_CXCR4_@OMV could bind to both sizes of gold nanoparticles simultaneously, indicating that mBMSC_CXCR4_@OMV was a true conjugate of mBMSC_CXCR4_ and OMV (Figure S3, Supporting Information). The stability of mBMSC_CXCR4_@OMV nanovesicles was investigated in PBS for over 1 week, and no signs of aggregation were detected (Figure 1H), thus indicating their excellent aqueous stability. The surface zeta potentials of all mBMSC_CXCR4_, OMV, and mBMSC_CXCR4_@OMV were negative, with the zeta potential of mBMSC_CXCR4_@OMV falling within the range observed for both mBMSC_CXCR4_ and the OMV (Figure 1I). Additionally, live and dead staining and quantitative analysis following treatment with PBS, mBMSC_CXCR4_, OMV, and mBMSC_CXCR4_@OMV for 3 days indicated no significant difference in cell viability of normal BMSCs and HUVECs (Figure S4, Supporting Information). Together, these data demonstrated that the prepared mBMSC_CXCR4_@OMV nanovesicles were successfully constructed with outstanding physicochemical features and good biocompatibility, thus making them appropriate for further biomedical investigations.

### 2.2 mBMSC_CXCR4_@OMV Selectively Accumulates in the Bone Marrow and Modulates Bone Marrow‐Derived Macrophages

**Figure 2:**
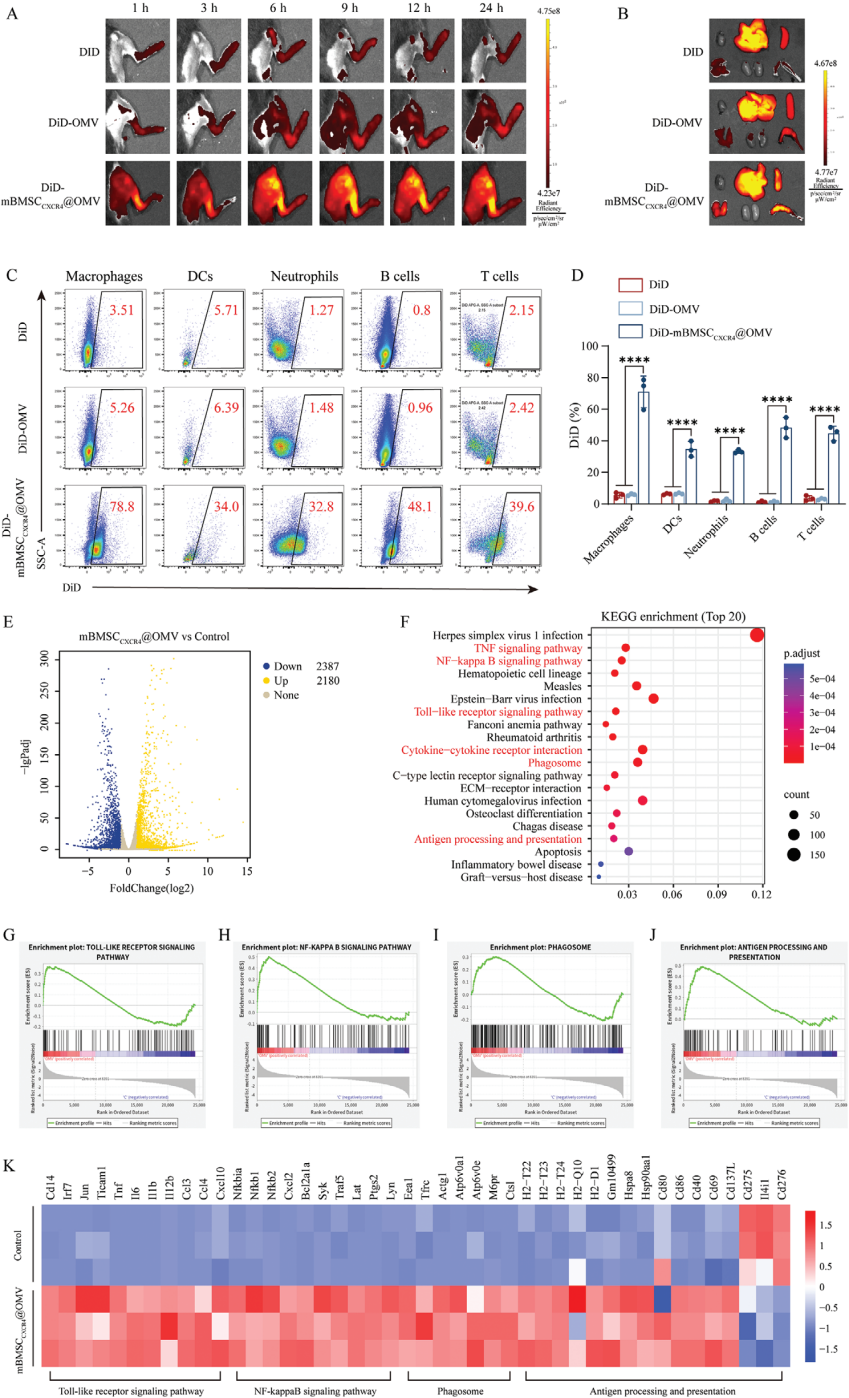
mBMSC_CXCR4_@OMV selectively accumulates in the bone marrow and modulates BMDM. A) Representative fluorescence images of mice under different treatment at 1, 3, 6, 9, 12, and 24 h after intravenous injection. B) Representative fluorescence images of bone marrow and major organs taken at 9 h post‐injection of DiD‐labeled nanovesicles. C) Flow cytometry analysis of the cellular uptake of DiD‐labeled nanovesicles in the bone marrow. D) The percent of DiD‐positive cells in macrophages, DCs, neutrophils, B cells, and T cells extracted from the bone marrow. E) Volcano plots showing the gene expression levels in Control and mBMSC_CXCR4_@OMV treatment groups. F) KEGG enrichment analysis showing top 20 statistically significant pathways regulated by mBMSC_CXCR4_@OMV. G–J) The GSEA results of Toll‐like receptor, NF‐kappa B, Phagosome, and Antigen processing and presentation signaling pathway. K) Heat map of genes related to immune response and phagocytosis. All values are expressed as the mean ± SD, with *n* = 3 independent samples. Statistical significance was determined using one‐way ANOVA. ^*^ *p* < 0.05, ^**^ *p* < 0.01, ^***^ *p* < 0.001, ^****^ *p* < 0.0001, and n.s. indicates no significance.

Bone marrow harbors abundant immune cells and is considered an important immunomodulatory organ for tumors and infections.^[^ ^28^, ^29^, ^30^ ^]^ To date, bone marrow has received increasing attention as a target for immunotherapy,^[^ ^40^, ^42^ ^]^ and targeting the bone marrow to activate the local immune response can effectively avoid systemic inflammatory storms. This is expected to become a breakthrough in bone‐related infection treatment. To confirm the bone marrow‐homing ability of mBMSC_CXCR4_@OMV, implant‐associated femoral osteomyelitis murine models created using C57 mice were intravenously injected with free DiD, DiD‐labeled OMV (DiD‐OMV), and DiD‐labeled mBMSC_CXCR4_@OMV (DiD‐mBMSC_CXCR4_@OMV). Strong fluorescence signals of DiD‐mBMSC_CXCR4_@OMV in the infected lower limbs were observed at 1 h after injection, whereas weak fluorescence signals of DiD and DiD‐OMV were observed. The fluorescence signal of DiD‐mBMSC_CXCR4_@OMV in the bone marrow of the infected lower limbs gradually increased over time, reaching a peak at 9 h after injection (**Figure** **2**A). At 24 h after intravenous injection, fluorescent signals in the bone marrow and major organs were detected ex vivo. It was clearly demonstrated that DiD, DiD‐OMV, and DiD‐mBMSC_CXCR4_@OMV primarily accumulated in the liver, spleen, and lung. Additionally, compared to the detectable signals of free DiD and DiD‐OMV, much stronger fluorescence was detected in the bone marrow by DiD‐mBMSC_CXCR4_@OMV, implying the targeting capability of DiD‐mBMSC_CXCR4_@OMV to the infected bone marrow after intravenous injection (Figure 2B). The difference in the targeted accumulation between DiD‐OMV and DiD‐mBMSC_CXCR4_@OMV suggested that fusion with mBMSC_CXCR4_ to form mBMSC_CXCR4_@OMV could overcome the immune clearance problem of OMVs and enabled effective bone marrow homing. In particular, bone marrow is a pivotal lymphoid organ that contains diverse immune cell subsets and plays an indispensable role in the fight against infections. To further assess the cellular uptake of mBMSC_CXCR4_@OMV in the infected bone marrow, flow cytometry was performed at 9 h after intravenous injection. According to these results, mBMSC_CXCR4_ inclusion enhanced cellular uptake in the bone marrow (Figure 2C,D). Notably, >70% of the BMDM were DiD‐positive after injection with DiD‐mBMSC_CXCR4_@OMV, and this was higher than other DiD‐positive DCs, neutrophils, B cells, and T cells extracted from the bone marrow (Figure 2C,D), indicating that macrophages were more likely to engulf mBMSC_CXCR4_@OMV in the infected bone marrow.

Macrophages, as essential components of the innate immune system, can be switched to either the pro‐inflammatory M1 or anti‐inflammatory M2 phenotype.^[^ ^43^, ^44^, ^45^ ^]^ M1 macrophages recognize, engulf, and destroy bacteria, and then present bacterial antigens to adaptive immune cells that are integral to the host defense against invading pathogens. The induction of macrophage M1 polarization is an effective strategy for eliminating bacterial infections. Given the observation that macrophages are more prone to engulf mBMSC_CXCR4_@OMV, we further explored the genomic changes of macrophages affected by mBMSC_CXCR4_@OMV. RNA sequencing of murine RAW264.7 macrophages was performed to map the genomic landscape. A total of 4558 differentially expressed genes (DEGs) were identified, of which 2180 were upregulated and 2387 were downregulated in the mBMSC_CXCR4_@OMV‐treated group (Figure 2E). Kyoto Encyclopedia of Genes and Genomes (KEGG) and Gene Set Enrichment Analysis (GSEA) analysis revealed that mBMSC_CXCR4_@OMV treatment activated the pathways related to M1 polarization, innate immunity, and cytokine production as evidenced by enrichment of the Toll‐like receptor (TLR) signaling pathway, the nuclear factor kappa‐B (NF‐κB) signaling pathway, the TNF signaling pathway, and the cytokine‐cytokine receptor interaction (Figure 2F,J). The expression levels of the pro‐inflammatory cytokines tumor necrosis factor (TNF), Interleukin‐6 (IL‐6), IL‐1β, and IL‐12β, and the chemokines CCL3, CCL4, CXCL10, and CXCL2 were observed to be upregulated (Figure 2K). The NF‐κB nuclear transposition driven by the TLR signaling pathway that leads to the increased transcription of inflammatory cytokines has been confirmed to be a key event in M1 macrophage polarization. These findings indicated that mBMSC_CXCR4_@OMV successfully polarized macrophages to the M1 phenotype by activating TLR/NF‐κB signaling pathway. Moreover, pro‐inflammatory M1 macrophages kill bacteria by phagocytosis and secretion of inflammatory cytokines. KEGG and GSEA analyses demonstrated that DEGs were enriched in phagosomes (Figure 2F,I), and the expression levels of genes related to phagocytosis such as Eea1, Tfrc, Actgg1, Atp6v0a1, Atp6v0e, M6pr, and Ctsl were upregulated (Figure 2K), indicating that mBMSC_CXCR4_@OMV enhanced the innate immunity of pro‐inflammatory M1 macrophages to eliminate bacteria.

Initial adaptive immunity requires the involvement of a dual signaling system, including MHC and immune co‐stimulatory molecules.^[^ ^10^, ^46^, ^47^ ^]^ In addition to the first signal provided by the MHC antigenic peptide, the second signal provided by costimulatory molecules is essential for activating adaptive immunity. Immune co‐activator molecules enable macrophages to enhance the activation of B and T cells and enhance adaptive immunity, whereas immune‐inhibitory molecules regulate the duration and amplitude of B and T cell responses, thereby hindering adaptive immunity.^[^ ^48^, ^49^ ^]^ These DEGs were associated with antigen processing and presentation (Figure 2F,J). The expression of MHC molecules such as H2‐T22, H2‐T23, H2‐T24, H2‐Q10, and H2‐D1 was significantly elevated in cells treated with mBMSC_CXCR4_@OMV (Figure 2K). Notably, mBMSC_CXCR4_@OMV upregulated the expression of co‐activator molecules, including CD86, CD40, CD69, and CD137L. In contrast, the expression of the immune‐inhibitory molecules CD275, CD276, and IL4i1 was significantly decreased in mBMSC_CXCR4_@OMV‐treated cells (Figure 2K). Collectively, these findings suggested that mBMSC_CXCR4_@OMV promoted the antigen‐presenting ability of macrophages, thereby synergistically activating adaptive immune responses.

### 2.3 mBMSC_CXCR4_@OMV Induces Macrophages to M1 Phenotype and Enhances Bactericidal Capacity In Vitro

**Figure 3:**
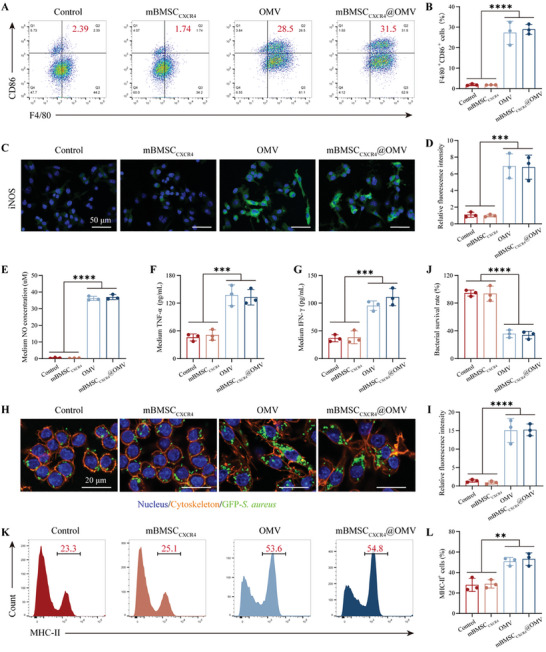
mBMSC_CXCR4_@OMV induces macrophages to M1 phenotype and enhances bactericidal capacity in vitro. A) Representative flow cytometry plots displaying changes in F4/80 and CD86 expression in RAW264.7 treated with PBS, mBMSC_CXCR4_, OMV, and mBMSC_CXCR4_@OMV. B) Quantification of M1 macrophages (F4/80^+^CD86^+^). C) Immunofluorescence staining of RAW264.7 for iNOS. D) Quantification of the relative fluorescence intensity of iNOS. E–G) The concentration of NO, TNF‐α, and IFN‐γ in the medium of RAW264.7 after different treatments. H) Fluorescence staining showing the bacterial phagocytosis of RAW264.7. I) Quantification of the relative fluorescence intensity of phagocytic bacteria. J) The survival rate of bacteria incubated with RAW264.7 after different treatments. K) Representative flow cytometry plots displaying changes in MHC‐II expression in RAW264.7. L) Quantification of MHC‐II^+^ cells. All values are expressed as the mean ± SD, with *n* = 3 independent samples. Statistical significance was determined using one‐way ANOVA. ^*^ *p* < 0.05, ^**^ *p* < 0.01, ^***^ *p* < 0.001, ^****^ *p* < 0.0001, and n.s. indicates no significance.

Based on the RNA sequencing results, we further validated the polarization status, phagocytosis, bactericidal capacity, and antigen presentation of macrophages treated with mBMSC_CXCR4_@OMV. M1 macrophages overexpress CD86, produce nitric oxide (NO), and release pro‐inflammatory cytokines to defend against foreign pathogens.^[^ ^12^, ^50^ ^]^ The RAW264.7 cells were incubated with mBMSC_CXCR4_@OMV nanovesicles to assess their potential to initiate macrophage differentiation. A dramatic increase in M1 macrophages (F4/80^+^CD86^+^ cells) was observed in RAW264.7 cells treated with OMV and mBMSC_CXCR4_@OMV (**Figure** **3**A,B). The expression of M1 phenotype marker inducible nitric oxide synthase (iNOS) was upregulated in OMV‐treated and mBMSC_CXCR4_@OMV‐treated RAW264.7 cells (Figure 3C,D). The concentration of NO was detected, and the results were consistent with the levels of iNOS (Figure 3E). Enzyme‐Linked Immunosorbent Assay (ELISA) assay was also implied to assess the release of tumor necrosis factor‐α (TNF‐α) and interferon‐γ (IFN‐γ) (pro‐inflammatory cytokines) in the medium, and OMV and mBMSC_CXCR4_@OMV treatment significantly elevated the levels of these cytokines compared to those in the control and mBMSC_CXCR4_ groups (Figure 3F,G). Subsequently, we assessed the phagocytic and bactericidal capacities of the macrophages. The RAW264.7 cells were pretreated with PBS, mBMSC_CXCR4_, OMV, or mBMSC_CXCR4_@OMV and then co‐incubated with GFP‐labeled *S. aureus*. Macrophages treated with OMV or mBMSC_CXCR4_@OMV engulfed more *S. aureus* than did the control and mBMSC_CXCR4_ groups (Figure 3H,I). Furthermore, macrophages polarized with OMV or mBMSC_CXCR4_@OMV exerted the most significant killing effect on *S. aureus*, as evidenced by reduced bacterial colony counts and scanning electron microscope (SEM) (Figure 3J; Figure S7, Supporting Information). Moreover, we examined the expression of MHC‐II, a protein that plays an important role in presenting antigen fragments to B and T cells to initiate an adaptive immune response, in RAW264.7 cells under different treatments. The results demonstrated that the numbers of MHC‐II‐positive cells were markedly increased in the OMV and mBMSC_CXCR4_@OMV groups (Figure 3K,L). In summary, mBMSC_CXCR4_@OMV polarized M0 macrophages to the M1 phenotype, enhanced phagocytosis and bactericidal capacity, and promoted bacterial antigen presentation, and this was consistent with the RNA sequencing results.

### 2.4 mBMSC_CXCR4_@OMV Potentiates Primary IAIs Regression

**Figure 4:**
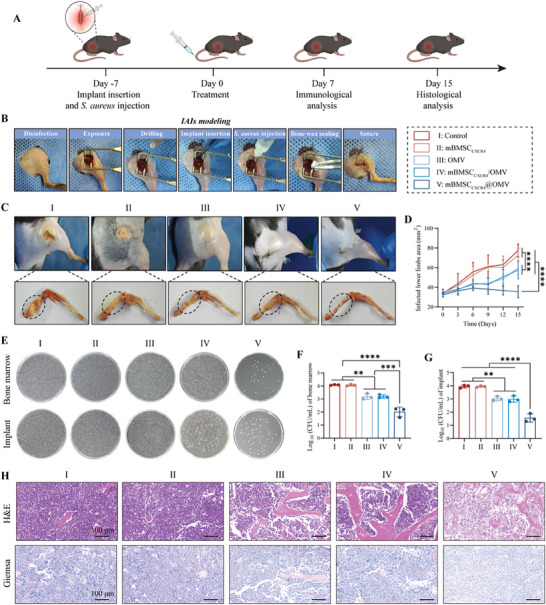
mBMSC_CXCR4_@OMV potentiates primary IAIs regression in vivo. A) Schematic diagram of the construction and intervention of the murine implant‐related femoral osteomyelitis. B) The surgical process for modeling implant‐related femoral osteomyelitis. C) Digital photos of the infected femur 15 days after treatment (n = 6). D) Average infected lower limbs area during treatment (n = 6). E–G) *S. aureus* CFU counts in the infected bone marrow and extracted implant (n = 3). H) H&E and Giemsa staining images of infected bone marrow (n = 3). Scale bar: 100 µm. All values are expressed as the mean ± SD. Statistical significance was determined using one‐way ANOVA. ^*^ *p* < 0.05, ^**^ *p* < 0.01, ^***^ *p* < 0.001, ^****^ *p* < 0.0001, and n.s. indicates no significance.

Given the excellent immune activation and anti‐bacterial properties of mBMSC_CXCR4_@OMV in vitro, we further validated its therapeutic efficacy in vivo. **Figure** **4**A illustrates the entire process of murine model construction, treatment time points, and efficacy observations. First, implant‐related femoral osteomyelitis mouse models with *S. aureus* infection were generated according to the standard surgical process presented in Figure 4B. 7 days after *S. aureus* injection, the mice were randomly divided into 5 groups (control, mBMSC_CXCR4_, OMV, mBMSC_CXCR4_/OMV, and mBMSC_CXCR4_@OMV), and diverse treatments were intravenously administered. The infected lower limbs were closely monitored within 15 days after receiving different treatments. Specifically, the infected area of lower limbs in the control saline and mBMSC_CXCR4_ groups continued to increase sharply, whereas the OMV‐and mBMSC_CXCR4_/OMV‐treated groups displayed a relatively moderate increase. Compared to the above groups, mBMSC_CXCR4_@OMV‐treated mice exhibited superior infection control as revealed by a gradual decline in the infected area. Consistently, mBMSC_CXCR4_@OMV‐treated mice indicated a slightly swollen area in the lower limbs without apparent signs of infections and exhibited the most rapid recovery, whereas varying degrees of severe swelling and purulent lower limbs developed in the other 4 groups (Figure 4C,D). Although the OMV‐and mBMSC_CXCR4_/OMV‐treated groups still demonstrated a significant decrease in the infected area compared to that of the control saline and mBMSC_CXCR4_ groups, the curative effect of free OMV without hybridization was unsatisfactory due to insufficient bone marrow targeting, resulting in insufficient uptake by the infected focus. During the therapeutic process, the body weights of the control and mBMSC_CXCR4_ groups tended to consistently decrease to some extent due to severe infection, whereas that of the mBMSC_CXCR4_@OMV treatment group remained quite stable, reflecting the considerable inhibition of infection (Figure S8, Supporting Information).

The bone marrow and implants were also harvested, and the relevant colony count assay was further utilized. Compared to the other 4 groups, the mBMSC_CXCR4_@OMV‐treated group indicated a superior reduction in bacterial colonies (Figure 4E,G). Moreover, hematoxylin‐eosin (H&E) staining revealed varying degrees of severe inflammatory responses in the control, mBMSC_CXCR4_, OMV, and mBMSC_CXCR4_/OMV groups, including a large number of lymphocytes, monocytes, and neutrophils, whereas inflammatory cell infiltration in the mBMSC_CXCR4_@OMV group was markedly reduced (Figure 4H). Similarly, Giemsa staining revealed substantial bacterial infiltration in the control, mBMSC_CXCR4_, OMV, and mBMSC_CXCR4_/OMV groups, whereas near‐complete elimination was observed in the mBMSC_CXCR4_@OMV group (Figure 4H), indicating satisfactory eradication of implant‐related femur osteomyelitis by mBMSC_CXCR4_@OMV. Additionally, serum biochemical analysis at the end of treatment indicated that aspartate aminotransferase (AST), alanine aminotransferase (ALT), urea nitrogen (UREA), creatinine (CREA), total protein (TP), and total bilirubin (TBIL) levels were within the normal range (Figure S9, Supporting Information), and H&E staining demonstrated no significant pathology in the murine organs (heart, liver, spleen, lung, and kidney) (Figure S10, Supporting Information), additionally illustrating high tissue compatibility and biosafety of mBMSC_CXCR4_@OMV treatment.

### 2.5 mBMSC_CXCR4_@OMV Boosts Innate and Adaptive Immune Response

**Figure 5:**
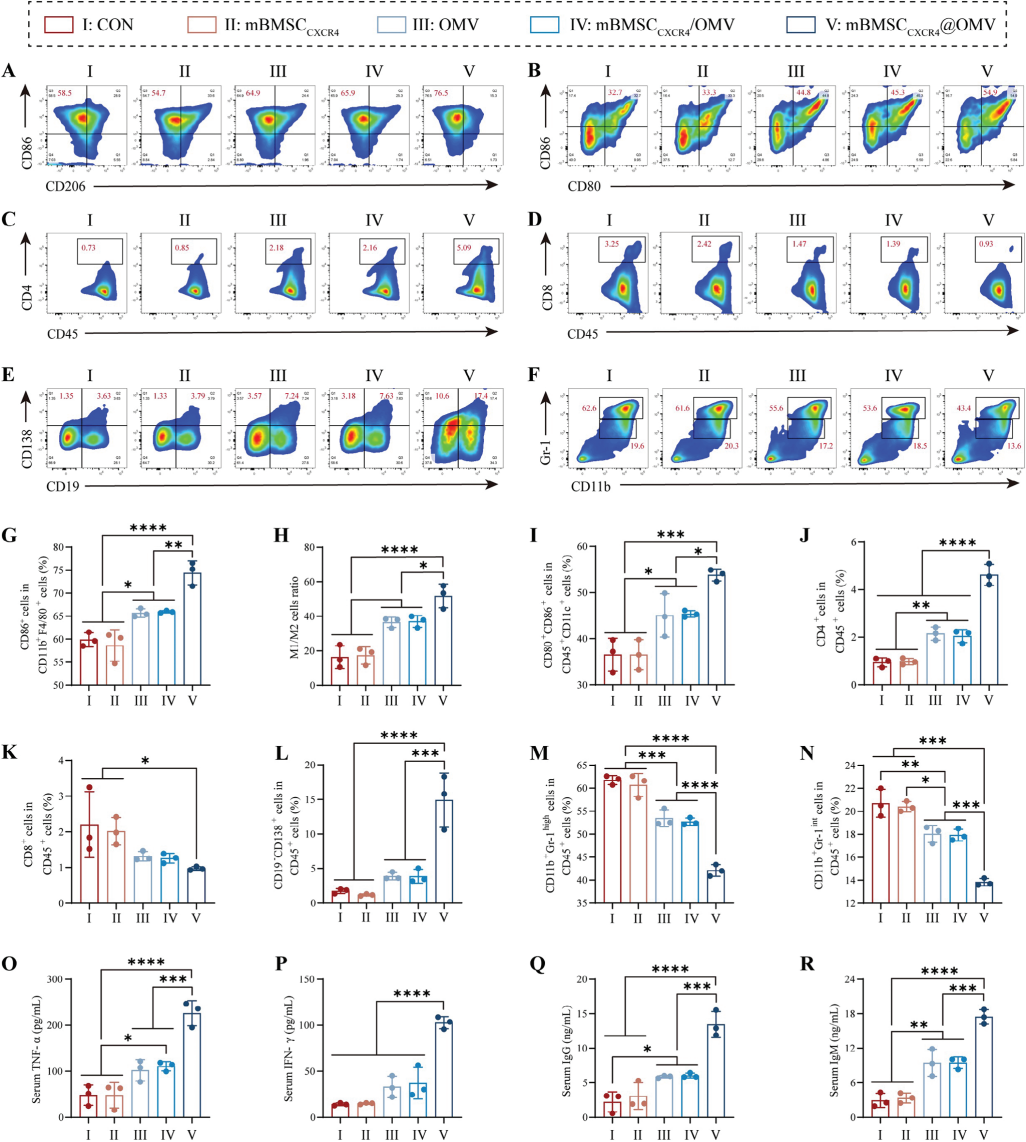
mBMSC_CXCR4_@OMV boosts innate and adaptive immune response. A–F) Representative flow cytometry plots of immune cells in the bone marrow on day 7 after treatment. A) M1 macrophages (CD86^+^) after gating on CD45^+^CD11b^+^F4/80^+^ cells. B) Mature DCs (CD80^+^CD86^+^) after gating on CD45^+^CD11c^+^ cells. C) CD4^+^ T cells (CD4^+^) after gating on CD45^+^ cells. D) CD8^+^ T cells (CD8^+^) after gating on CD45^+^ cells. E) Plasma cells (CD19^−^CD138^+^) after gating on CD45^+^ cells. F) Granulocytic MDSCs (CD11b^+^Gr1^high^) and monocytic MDSCs (CD11b^+^Gr1^int^) after gating on CD45^+^ cells. G) Quantification of M1 macrophages. H) The ratio of M1 to M2 macrophages. I) Quantification of mature DCs. J) Quantification of CD4^+^ T cells. K) Quantification of CD8^+^ T cells. L) Quantification of plasma cells. M) Quantification of granulocytic MDSCs. (N) Quantification of monocytic MDSCs. O–R) The serum concentration of TNF‐α, IFN‐γ, IgG, and IgM. All values are expressed as the mean ± SD, with *n* = 3 independent samples. Statistical significance was determined using one‐way ANOVA. ^*^ *p* < 0.05, ^**^ *p* < 0.01, ^***^ *p* < 0.001, ^****^ *p* < 0.0001, and n.s. indicates no significance.

Furthermore, the potency of mBMSC_CXCR4_@OMV for regulating the bone marrow's immune microenvironment (IME) was evaluated in vivo. Flow cytometry was performed on day 7 post‐injection, and the results revealed strong anti‐bacterial immune responses in the locally infected bone marrow IME. As illustrated by our in vivo uptake data, the mBMSC_CXCR4_@OMV nanovesicles could be specifically delivered to the bone marrow and effectively taken up by bone marrow‐derived macrophages. Pro‐inflammatory M1 macrophages (CD45^+^CD11b^+^F4/80^+^CD86^+^) were significantly increased in the mBMSC_CXCR4_@OMV group compared to that of the control, mBMSC_CXCR4_, OMV, and mBMSC_CXCR4_+OMV groups (**Figure** **5**A,G). In particular, compared to the control group in the bone marrow IME, the ratio of M1 to M2 in the mBMSC_CXCR4_@OMV group was upregulated >3‐fold (Figure 5A,H). Subsequent investigations regarding the levels of TNF‐α and IFN‐γ confirmed that mice treated with mBMSC_CXCR4_@OMV exhibited superior bactericidal innate immunity (Figure 5O,P). Research has demonstrated that M1 macrophages engulf and eradicate the pathogens in the early antibacterial microenvironment; meanwhile, they also secrete a variety of chemokines, which facilitate the recruitment of BMSCs, osteoprogenitor cells, and vascular progenitor cells to the site of injury.^[^ ^51^ ^]^ With the elimination of bacteria and the inflammatory process subsides, subsequent transition to anti‐inflammatory phenotype further initiates bone repair and regeneration.^[^ ^52^ ^]^ The superior bactericidal innate immunity suggested that nanovesicle mBMSC_CXCR4_@OMV contributes to the further management of implant‐related complications. Moreover, an elevated number of M1 macrophages promote antigen presentation, thereby triggering adaptive immunity. Additionally, we observed that the proportion of mature DCs (CD45^+^CD11c^+^CD80^+^CD86^+^) that are considered another important type of APCs was remarkably upregulated in the IME of mBMSC_CXCR4_@OMV‐treated mice (Figure 5B,I). Collectively, the immunosuppressive microenvironment of bacterial infection is characterized by exhausted APCs, whereas these results confirmed the status transition of M1 macrophages and DCs maturation, suggesting that mBMSC_CXCR4_@OMV facilitated a pronounced enhancement of bacterial antigen presentation.

T cells are a vital component of adaptive immunity. When the antigen receptor complex of T cells encounters peptide antigens presented by APCs, it can be activated via direct cell‐to‐cell interactions.^[^ ^53^, ^54^, ^55^, ^56^ ^]^ We hypothesized that an enhanced population of M1 macrophages and mature DCs in the bone marrow IME triggers adequate adaptive anti‐bacterial immunity. Encouragingly, greater numbers of CD4^+^ T cells were observed in the mBMSC_CXCR4_@OMV‐treated mice, while a slight reduction in the proportion of CD8^+^ T cells was detected compared to levels in the other 4 groups (Figure 5C,J). Additionally, the ratio of CD4^+^ to CD8^+^ T cell in mBMSC_CXCR4_@OMV‐treated mice increased by >9‐fold compared to that in the control (Figure S11, Supporting Information), and this was likely owing to more CD4^+^ T cells activation. As previously reported, enhanced infiltration of CD4^+^ T cells may indicate more powerful B cell‐triggered anti‐bacterial immunity.^[^ ^10^, ^53^ ^]^ With the assistance of CD4^+^ T cells, antigen‐primed B‐lymphocytes clonally expand, and thus differentiate into antibody‐secreting plasmablasts and plasma cells upon infection.^[^ ^54^ ^]^ Compared to the control group, mBMSC_CXCR4_@OMV generated >3‐ and 8‐fold increases in the proportion of plasmablasts (CD45^+^CD19^+^CD138^+^) and plasma cells (CD45^+^CD19^−^CD138^+^) in the infected bone marrow, respectively (Figure 5E,L; Figure S12, Supporting Information). Especially, the greater numbers of long‐life plasmablasts represent a more durable source of specific antibodies. Additionally, the administration of mBMSC_CXCR4_@OMV elevated the titers of serum IgG and IgM antibodies (Figure 5Q,R), suggesting that mBMSC_CXCR4_@OMV was effective for inducing B cell‐mediated humoral immunity.

Moreover, massive infiltration of immunoinhibitory MDSCs into the bone marrow represents a hallmark of the immunosuppressive microenvironment.^[^ ^57^, ^58^ ^]^ To quantify the effect of mBMSC_CXCR4_@OMV on immunomodulation, MDSCs infiltration in the bone marrow was subsequently analyzed. Not surprisingly, an obvious reduction of both granulocytic MDSCs (CD45^+^CD11b^+^Gr1^high^) and monocytic MDSCs (CD45^+^CD11b^+^Gr1^int^) was observed in the mBMSC_CXCR4_@OMV group (Figure 5M,N). As the massive infiltration of immunoinhibitory MDSCs lead to the inactivation of T cells and block the effector functions of immunocyte, mBMSC_CXCR4_@OMV‐triggered MDSCs depletion promoted the efficacy of anti‐bacterial immune response. In summary, mBMSC_CXCR4_@OMV boosted both innate and adaptive immunity against murine IAIs. Specifically, mBMSC_CXCR4_@OMV polarized macrophages into the M1 phenotype with excellent bactericidal capability, promoted DC maturation, presented bacterial antigens together with M1 macrophages, and achieved adaptive immunity triggered by APC‐activated T cell immunity and B cell‐mediated humoral immunity.

### 2.6 mBMSC_CXCR4_@OMV Prevents IAIs Relapse

In the clinical practice, the standard treatment for chronic or recurrent IAIs is revision surgery plus antibiotics.^[^ ^1^, ^2^ ^]^ However, revision surgeries, including stage I and stage II revisions, are still challenging based on high rates of relapse and reinfection because of inadequate elimination of infected tissues, antibiotic resistance, and bacterial deposits.^[^ ^3^, ^4^, ^5^ ^]^ Antigenic hypervariability and microbiome heterogeneity of bacterial infections result in a constricted range of bacteria‐specific immunological memory responses.^[^ ^14^, ^15^ ^]^ Particularly in certain low‐grade infections, it is difficult to generate sufficient immunogenicity to stimulate effective immune surveillance, resulting in negligible immune defense and the development of chronic infections or relapses. As recently reported, neoadjuvant immunotherapy holds promise for promoting the immune response before surgical operation to prevent postsurgical relapse.^[^ ^15^, ^53^ ^]^ Encouraged by the remarkable therapeutic effect of mBMSC_CXCR4_@OMV for primary implant‐related femoral osteomyelitis, we hypothesized that mBMSC_CXCR4_@OMV as a neoadjuvant immunotherapy before revision surgery could enhance immune responses to boost humoral immune memory in the bone marrow IME to prevent relapse after revision surgery.

**Figure 6:**
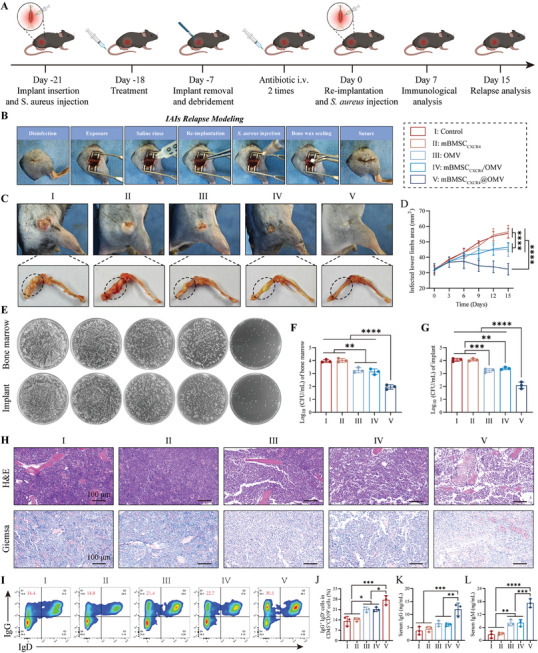
mBMSC_CXCR4_@OMV prevents IAIs relapse. A) Schematic diagram of the construction and treatment of the implant‐related femoral osteomyelitis relapse. B) The surgical process for modeling implant‐related femoral osteomyelitis relapse. C) Digital photos of the infected femur 15 days after relapse (n = 6). D) Average infected lower limbs areas of the relapse infection (n = 6). E–G) *S. aureus* CFU counts in the re‐infected bone marrow and extracted implant (n = 3). H) H&E and Giemsa staining images of re‐infected bone marrow (n = 3). Scale bar: 100 µm. I) Representative flow cytometry plots of memory B cells (IgG^+^IgD^−^) after gating on CD45^+^CD19^+^ cells in the bone marrow on day 7 after relapse (n = 3). J) Quantification of memory B cells (n = 3). K,L) The serum concentration of IgG and IgM (n = 3). All values are expressed as the mean ± SD. Statistical significance was determined using one‐way ANOVA. ^*^ *p* < 0.05, ^**^ *p* < 0.01, ^***^ *p* < 0.001, ^****^ *p* < 0.0001, and n.s. indicates no significance.

Therefore, the primary implant‐related femoral osteomyelitis mouse model was established on day ‐21, and the mice were treated with saline, mBMSC_CXCR4_, OMV, mBMSC_CXCR4_/OMV, or mBMSC_CXCR4_@OMV on day ‐18. The implants were removed, the infectious sites were thoroughly debrided on day ‐7, and vancomycin was administered twice intravenously, mimicking the clinical revision surgical procedures for IAIs. Subsequent re‐insertion of new implants and injection of *S. aureus* into the femur was implemented to construct a recurrent femoral osteomyelitis model to assess the defense against relapse (**Figure** **6**A,B; Figure S13, Supporting Information). Compared to the other 4 groups with higher relapse rates, mice treated with mBMSC_CXCR4_@OMV withstood the bacterial rechallenge and exhibited largely alleviated lower‐limb swelling. Ex vivo data from harvested femurs also demonstrated that mBMSC_CXCR4_@OMV treatment led to sharply decreased infection severity without discharge of pus, while the control and mBMSC_CXCR4_ groups exhibited severe purulent performance with visible bone fracture and similar obvious infected symptoms in the OMV and mBMSC_CXCR4_/OMV groups (Figure 6C,D). Consistently, the bacterial colonies cultured from the harvested bone marrow and implants demonstrated a superior decline in the mBMSC_CXCR4_@OMV group as compared to the other 4 groups (Figure 6E–G). H&E and Giemsa staining images validated the high resistance of mBMSC_CXCR4_@OMV to relapse after revision surgery (Figure 6H). Flow cytometry using harvested bone marrow revealed that the mBMSC_CXCR4_@OMV‐treated group elicited large pools of memory B cells (Figure 6I,J). Additionally, serum IgG and IgM levels significantly enhanced in the mBMSC_CXCR4_@OMV group than any other groups (Figure 6K,L), indicating that the durable antibody‐secreting potential of mice treated with mBMSC_CXCR4_@OMV may be the key point for relapse control. Taken together, these results highlight the effectiveness of mBMSC_CXCR4_@OMV as a neoadjuvant immunotherapy to induce long‐term immune memory to prevent the relapse of infection, holding great promise for reducing antibiotic use and surgical frequency in IAIs treatment.

## 3 Conclusion

In summary, an immune‐strengthening nanovesicle, mBMSC_CXCR4_@OMV, is constructed by the hybridization of *E. coli‐*derived OMVs and mBMSC_CXCR4_ isolated from genetically engineered BMSCs overexpressing CXCR4. In particular, the mBMSC_CXCR4_@OMV nanovesicle selectively accumulates in the infected bone site and is effectively taken up by the bone marrow‐derived macrophages, triggering the efficient transition to pro‐inflammatory M1 status through TLR/NF‐κB pathway. This alteration promotes its innate bactericidal capacity and antigen presentation as APC. Subsequent activation of T and B cells and inhibition of MDSCs in the bone marrow facilitates in vivo clearance of infections through adaptive immunity in a mouse model. Additionally, mBMSC_CXCR4_@OMV boosts memory B cells and bacteria‐specific antibody responses. Taken together, this study warrants mBMSC_CXCR4_@OMV to eradicate complicated IAIs and provide whole‐stage protection against postsurgical relapse, marking a significant immunotherapeutic advancement in the post‐antibiotic era.

## 4 Experimental Section

### Preparation of OMVs

The OMVs were obtained according to the previous reports.^[^ ^18^, ^20^, ^21^ ^]^ Briefly, the engineered *E. coli* were cultured in Luria‐Bertani (LB) medium at 37 °C with agitation at 220 rpm until reaching an optical density at 600 nm (OD600) of 0.6. Afterward, isopropyl β‐D‐1‐thiogalactopyranoside (IPTG) at a concentration of 0.1 mm was added to induce protein expression, and the temperature was reduced to 16 °C for a further 14 h incubation period with shaking at 160 rpm. After IPTG induction, the cell supernatant was obtained by centrifugation at 6000 rpm for 10 min, and subsequently filtered through a 0.45 µm membrane. The filtrate was concentrated using an ultrafiltration tube with a 100 kDa molecular weight cutoff. The concentrate was filtered again using a filter with 0.22 µm pore size, and was pelleted by ultracentrifugation at 150 000 g for 3 h at 4 °C. The resulting pellets were resuspended in 400 µL of PBS and stored at −80 °C for future use. The total protein concentration of the OMV preparations was determined by the bicinchoninic acid (BCA) method.

### Preparation of mBMSC_CXCR4_

Plasmid construction was briefly described as follows. The gene encoding CXCR4 (NCBI Accession No. 0 014 20068.1) was synthesized by GENEWIZ (Suzhou, China). The detailed amino acid and DNA sequences for CXCR4 are provided in Table S1 (Supporting Information). The gene fragments were then subcloned into the pLV3‐EF1α‐Puro vector, resulting in the construct pLV3‐EF1α‐CXCR4‐Puro. The vectors were subsequently transformed into *E. coli* BL21 (DE3) following the manufacturer's instructions (Tiangen Biotech Co., Ltd., Beijing, China) and stored at −80 °C for future use. BMSC_CXCR4_ stable expression cells were established using lentiviral particles produced by transfecting HEK 293T cells with lentiviral expression plasmid pLV3‐EF1a‐CXCR4‐Puro and packaging plasmids (pVSVg and psPAX2). Briefly, BMSCs were seeded in 6‐well plates, grown to 60–80% confluence, and then exposed to a medium containing the virus and 4 µg mL^−1^ polybrene (Santa Cruz Biotechnology, Dallas, TX). After infection for 48 h, the culture medium was refreshed to α‐MEM supplemented with 1.5 µg mL^−1^ puromycin (Thermo Fisher Scientific, Waltham, MA) for stable expression cell selection.

To isolate cell membranes from genetically engineered BMSC_CXCR4_ cells, the cultured cells were washed with PBS, scraped on ice using a cell scraper, and centrifuged at 1000 rpm and 4 °C for 5 min. The harvest cell pellet was resuspended in 4 mL of isolation buffer (containing 0.5 g of BSA, 0.5 mm EGTA, and 0.1 mm PMSF in a Tris‐HCl and sucrose solution, pH 7.4), then sonicated at 30 W for 5 min in an ice water bath. The sample was centrifuged at 3000 g and 4 °C for 5 min, and the supernatant was collected. This supernatant was further centrifuged at 10 000 × g and 4 °C for 10 min, and the resulting supernatant was collected. The final supernatant was subjected to ultracentrifugation at 100 000 g and 4 °C for 2 h. The pellet was resuspended in PBS, and membrane content was quantified using a BCA Kit. The isolated membranes were stored at −80 °C until further use.

### Hybridization of mBMSC_CXCR4_@OMV

The BMSC membranes and OMVs were first dispersed in ultrapure water at a mass ratio of 5:1, and then the mixed solutions underwent ultrasonication in a KQ‐300DE ultrasonic cleaner at 20 °C for 30 min (300 W). Afterward, the mixture was physically extruded ten times through a porous polycarbonate membrane (400 nm) using an Avanti mini extruder. Following extrusion, the solution was centrifuged at 12 000 rpm for 30 min at 4 °C to obtain mBMSC_CXCR4_@OMV.

### Characterization of mBMSC_CXCR4_, OMV, and mBMSC_CXCR4_@OMV

Carbon‐coated copper grids were used for TEM sample preparation, and they were discharged beforehand to enhance their hydrophilicity. And then, 5 µL of the sample solution was dropped onto the grid for 10 s, followed by removal of excess solution using filter paper. The samples were then stained with 5 µL of 1% uranyl acetate for 10 s. After removing the excess uranyl acetate solution and air‐drying for an additional 10 s, the morphology of the samples was observed under high vacuum using a TEM (JEM‐1230R, JEOL, Japan). The particle size and zata potential were measured using a nanoparticle tracking analysis instrument (NanoSight NS300, Malvern).

### Detection of Bone Marrow Cell Uptake of mBMSC_CXCR4_@OMV

DiD‐labeled mBMSC_CXCR4_@OMV were prepared for the cellular uptake of mBMSC_CXCR4_@OMV in the bone marrow. Implant‐related osteomyelitis murine models using C57BL/6 mice were intravenously injected with DiD, DiD‐OMV, or DiD‐mBMSC_CXCR4_@OMV. After 9 h, bone marrow cells were collected from the animals. Fixable Viability Stain 780 (FVS780) was used to rule out dead cells, and then bone marrow cells were stained with anti‐CD45‐FITC, anti‐CD11b‐PerCp‐Cy5.5, anti‐Ly‐6G‐PE, anti‐CD11c‐PE, anti‐CD3‐APC and anti‐CD19‐PE‐Cy7 antibodies (Table S2, Supporting Information). The proportions of DiD^+^CD11b^+^ macrophages, DiD^+^CD11b^+^Ly‐6G^+^ neutrophils, DiD^+^CD11c^+^ DCs, DiD^+^CD3^+^ T cells, and DiD^+^CD19^+^ B cells were detected by flow cytometry. The data were analyzed using the FlowJo software (V10).

### Evaluation of Immune Response In Vitro

To assess the polarization of RAW264.7 after different treatments, PBS, mBMSC_CXCR4_ (25 µg mL^−1^), OMV (25 µg mL^−1^), mBMSC_CXCR4_@OMV (50 µg mL^−1^) were incubated with RAW264.7 for 24 h. The supernatant was collected for NO (Beyotime, China) and cytokine (IFN‐γ, TNF‐α) analysis. For flow cytometry, the cells were washed twice with cold PBS and then stained with anti‐F4/80‐PE, anti‐CD86‐PE‐Cy7, anti‐MHC‐II‐FITC antibodies for 30 min at 4 °C (Table S3, Supporting Information). After being washed with Stain Buffer (FBS), cells were analyzed by flow cytometer (BD FACSMelody). For immunofluorescent staining, the cells were fixed with 4% paraformaldehyde for 10 min, and then blocked with Immunostaining Blocking Solution (Beyotime, China) for 30 min. Subsequently, cells were incubated with anti‐iNOS antibody (Abcam, USA) at 4 °C overnight. After removing the primary antibody, cells were incubated with Multi‐rAb CoraLite Plus 488‐Goat Anti‐Rabbit Recombinant Secondary Antibody (H + L) (Proteintech, China) at room temperature for 1 h. DAPI (Servicebio, China) were used to label the nuclei. Finally, cells were observed with a confocal microscope (ZEISS LSM 710, Germany).

### Animal Study

Male C57BL/6 mice were purchased from Beijing Weitong Lihua Experimental Animals and all mice were maintained in a specific pathogen‐free environment. The animal experiments were authorized by the Institutional Committee on the Ethics of Animal Experiments of Zhengzhou University (2023‐KY‐0433‐001, Zhengzhou, China). All of the animal studies complied with the Guide for the Care and Use of Laboratory Animals of the National Institutes of Health.

### Establishment of Primary IAIs Models

To establish a mouse model of implant‐associated femur osteomyelitis, a 0.5 mm unicortical bone defect was made on the dorsal side of right femoral mid‐shaft, and a sterilized stainless pin was inserted into the medullary cavity through the canal. Next, 20 µL of *S. aureus* solution at 1×10^6^ CFU/mL was injected into the intramedullary cavity through the defect. Finally, the bone defect was sealed with bone wax and the incision was closed with a 5‐0 suture. 7 days after *S. aureus* injection, the mice were randomly divided into 5 groups (control, mBMSC_CXCR4_, OMV, mBMSC_CXCR4_/OMV, and mBMSC_CXCR4_@OMV), and treatments above were intravenously administered.

### Evaluation of Therapeutic Effect in Primary IAIs Models

After establishment of implant‐associated femoral osteomyelitis mouse model, the infected lower limbs area was measured throughout treatment. General observations of the lower limbs were recorded using an optical camera. 7 days after treatment, partial mice were sacrificed to evaluate the immune status of bone marrow. The bone marrow cells were filtered on a 70 µm strainer to obtain the single‐cell suspension. The cell suspensions were first incubated with Fixable Viability Stain 780 to exclude dead cells, and then stained with anti‐CD45‐FITC, anti‐CD11b‐PerCP‐Cy5.5, anti‐F4/80‐PE, anti‐CD86‐PE‐Cy7, anti‐CD206‐APC, anti‐CD11c‐PE, anti‐CD80‐APC, anti‐CD3‐APC, abti‐CD4‐PE, anti‐CD8‐PerCP‐Cy5.5, anti‐CD19‐PE‐Cy7, anti‐138‐APC, anti‐Gr‐1‐APC (Table S3, Supporting Information). The samples were examined using a flow cytometer (BD FACSMelody, USA). The pro‐inflammatory cytokines (TNF‐α and IFN‐γ) and antibodies (IgG and IgM) in the serum were detected using ELISA kits. In addition, 15 days after treatment, the bone marrow and implants were extracted for bacterial CFU assay. The infected lower limbs were fixed and sectioned for H&E staining and Giemsa staining.

### Evaluation of Prevention Effect in Relapse IAIs Models

After establishment of implant‐associated relapse femoral osteomyelitis model, general observations and areas of the infected lower limbs were recorded. 7 days after the rechallenge surgery, the bone marrow cells were harvested and stained with anti‐CD45‐FITC, anti‐CD19‐PE‐Cy7, anti‐IgG‐PE, anti‐IgD‐APC (Table S4, Supporting Information) to detect the memory B cells. The levels of IgG and IgM in the serum were detected using ELISA kits. 15 days after the rechallenge surgery, bacterial CFU assay, H&E staining and Giemsa staining were conducted to assess the prevention effect.

### Evaluation of Immune Response In Vivo

For primary implant‐associated femoral osteomyelitis models, 7 days after infection, the mice were sacrificed for immune evaluation. Both ends of the femur were cut, and bone marrow suspensions were collected using an injector. The cell suspensions were then filtered on a 70 µm strainer to obtain the single‐cell suspension. For the analysis of immune cells composition, the cell suspensions were first incubated with Fixable Viability Stain 780 to exclude dead cells, and then stained with different combinations of flow cytometry antibodies, which included anti‐CD45‐FITC, anti‐CD11b‐PerCp‐Cy5.5, anti‐F4/80‐PE, anti‐CD86‐PE‐Cy7, anti‐CD206‐APC, anti‐CD19‐PE‐Cy7, anti‐CD138‐APC, anti‐CD3‐APC, anti‐CD4‐PE, anti‐CD8‐PerCp‐Cy5.5, anti‐CD11c‐PE, anti‐CD80‐APC, anti‐Gr‐1‐APC (Table S4, Supporting Information). The samples were examined using a flow cytometer (BD FACSMelody, USA). The cytokines (IFN‐γ, TNF‐α) and antibodies (IgG and IgM) in the serum were tested with ELISA kits according to the manufacturer's recommendations.

For relapse implant‐associated femoral osteomyelitis models, 7 days after re‐implantation, the mice were sacrificed for immune evaluation. The cell suspensions of bone marrow were collected using the above methods. To analyze the memory B cells, the cell suspensions were incubated with anti‐CD45‐FITC, anti‐CD19‐PE‐Cy7, anti‐IgD‐PE, and anti‐IgG‐APC (Table S4, Supporting Information). The samples were examined using a flow cytometer (BD FACSMelody, USA). The antibodies (IgG and IgM) in the serum were tested with ELISA kits according to the manufacturer's recommendations.

### Statistical Analysis

Statistical analysis was performed using GraphPad Prism 7.0 software. Quantitative data were performed for at least three times and the data were expressed as mean ± standard deviation (SD). Statistical significance was calculated via one‐way ANOVA and shown as follows: n.s. means no significance, ^*^ *p* < 0.05, ^**^ *p* < 0.01, ^***^ *p* < 0.001, and ^****^ *p* < 0.0001.

## Conflict of Interest

The authors declare no conflict of interest.

## Author Contributions

Z.W., M.L., and W.L. contributed equally to this work. All authors have given approval to the final version of the manuscript.

## Supporting information



Supporting Information

## Data Availability

The data that support the findings of this study are available from the corresponding author upon reasonable request.
