# Supplementary material for: Hybrid Outer Membrane Vesicles with Genetically Engineering for Treatment of Implant‐Associated Infections and Relapse Prevention Through Host Immunomodulation
Source: Adv Sci (Weinh). 2025 Feb 14;12(14):2415379. doi: 10.1002/advs.202415379 (PMC11984893; doi:10.1002/advs.202415379)
Supplement: Supplementary file 1 — Supporting Information [file ADVS-12-2415379-s001.docx]

**Supporting Information**

**Hybrid Outer Membrane Vesicles with Genetically Engineering for the Treatment of Implant-Associated Infections and Relapse Prevention Through Host Immunomodulation**

*Zhichao Wang^1#^, Mingfei Li^1,2#^, Wenshuai Li^2#^, Liuliang He^1^, Long Wang^1^, Kehan Cai^1^, Xiao Zhao^3^*, Yazhou Chen^2^*, and Daifeng Li^1^**

^1^ Department of Orthopedics, The First Affiliated Hospital of Zhengzhou University, Zhengzhou, 450052, China. Email address: lidaifeng@zzu.edu.cn

^2^ Medical 3D Printing Center, The First Affiliated Hospital of Zhengzhou University, Henan Institute of Advanced Technology of Zhengzhou University, Zhengzhou, 450052, China. Email address: yzchenbio@zzu.edu.cn

^3^ CAS Key Laboratory for Biomedical Effects of Nanomaterials and Nanosafety, CAS Center for Excellence in Nanoscience, National Center for Nanoscience and Technology of China, Beijing, 100190, China. Email address: zhaox@nanoctr.cn

^#^These authors contributed equally to this work.

*Correspondence:

Daifeng Li: lidaifeng@zzu.edu.cn

Yazhou Chen: yzchenbio@zzu.edu.cn

Xiao Zhao: zhaox@nanoctr.cn

**Materials and methods**

***Materials and reagents***

α-Minimum Essential Medium (α-MEM), Dulbecco’s modified Eagle medium (DMEM), exosome-depleted fetal bovine serum (FBS) and 1% penicillin-streptomycin were purchased from Thermo Scientific, USA. Flow cytometry antibodies, Fixable Viability Stain 780 were purchased from BD Bioscience Co. Ltd., UK. Cell strainer was purchased from Absin Bioscience Inc., China. Anti-iNOS antibody was purchased from Abcam, UK. NO assay kit, Actin-Tracker Red-Rhodamine, Immunostaining Blocking Solution, Isopropyl β-D-1-thiogalactopyranoside (IPTG) were purchased from Beyotime Biotechnology Co. Ltd., China. Fluorescent secondary antibody was purchased from Proteintech group Inc., China. IFN-γ, TNF-α, IL-6, IgG and IgM enzyme-linked immunisorbent assay (ELISA) kits were purchased from NeoBioscience Technology Co. Ltd., China. Penicillin-streptomycin and DAPI were purchased from Servicebio Biotechnology Co. Ltd., China. All other required chemicals were obtained from commercial sources and of analytical grade.

***Cell culture***

Bone marrow derived mesenchymal stem cells (BMSCs), and macrophage-like cells (RAW264.7) and mouse embryonic fibroblast cells (NIH3T3) were used in this work. The RAW264.7 were cultured in DMEM supplemented with 10% exosome-depleted FBS and 1% penicillin-streptomycin. BMSCs were cultured in α-MEM suspended with 10% FBS and 1% penicillin-streptomycin. NIH3T3 were cultured were cultured in DMEM containing high glucose supplemented with 10% FBS and 1% penicillin-streptomycin.

***SDS-PAGE and western blot analysis***

Protein expression was analyzed using SDS-PAGE and western blotting. Protein concentrations were quantified with the Pierce BCA Protein Assay Kit (Thermo Scientific, USA) following the manufacturer’s instructions. Equal amounts of total protein were separated by electrophoresis on 12% bis-Tris gels, transferred to nitrocellulose membranes, and probed with the primary anti-CXCR4 antibody (1 : 500, Cell Signaling Technology, 9678S). This was followed by incubation with a peroxidase-conjugated secondary antibody (Abcam). Immunoreactive bands were visualized using an ECL kit according to the manufacturer's guidelines. For normalization, membranes were reprobing with the Na^+^/K^+^ ATPase antibody (Cell Signaling Technology, 3010S) as an internal loading control.

***Co-localization analysis***

NIH3T3 cells were seeded into a confocal culture dish and allowed to grow overnight. The cells were then treated with DiI-labeled mBMSC_CXCR4_ (red fluorescence) and DiO-labeled OMV (green fluorescence). After 24 h, the supernatant was removed, and the cells were washed three times with PBS. Fluorescence distribution in the treated cells was observed using confocal laser scanning microscopy (CLSM) after staining the cell nuclei with Hoechst 33342. Colocalization analysis of the fluorescence in mBMSC_CXCR4_/OMV and mBMSC_CXCR4_@OMV was conducted using ImageJ software (NIH).

***Cell viability***

HUVECs and BMSCs were used for cell viability analysis. The cells were seeded onto a 96-well flat-bottom tissue culture plate (Corning, Falcon) at a density of 0.5 × 10^4^ cells/cm^2^ and incubated overnight to allow for cell attachment. The medium was then replaced with PBS, mBMSC_CXCR4_ (25 μg/mL), OMV (25 μg/mL), or mBMSC_CXCR4_@OMV (50 μg/mL), and cultured for 1, 2, and 3 days. For live/dead viability assessment, a subset of the samples was processed using calcein-AM and propidium iodide (PI) staining agents, obtained from the Live/Dead Double Staining Kit (Dojindo, Japan). After staining, cell viability was evaluated using fluorescence microscopy on a Nikon Eclipse Ts2R microscope, with live cells emitting green fluorescence and dead cells emitting red fluorescence. Cell viability was further quantitatively assessed using the WST-1 assay kit (Roche Diagnostics, IN) according to the manufacturer's instructions.

***Transcriptomic sequencing and analysis***

RAW264.7 cells were collected after treatment with PBS or mBMSC_CXCR4_@OMV (50 μg/mL). Total RNA was extracted with Trizol agent (Invitrogen, USA) for transcriptomic sequencing (Annoroad Gene Technology, Beijing, China). Fold change ≥ 2, p-value ≤ 0.05 and padj ≤ 0.05 was set as the threshold for significantly differential expression genes (DEGs). Heat map, Kyoto Encyclopedia of Genes and Genomes (KEGG) and Gene Set Enrichment Analysis (GSEA) were performed using SolarGenomic Cloud (Annoroad Gene Technology, Beijing, China).

***Phagocytosis and killing of S. aureus by RAW264.7***

RAW264.7 cells were pre-treated with either PBS, mBMSC_CXCR4_ (25 μg/mL), OMV (25 μg/mL) or mBMSC_CXCR4_@OMV (50 μg/mL) for 24 h, and then co-incubated with GFP-*S. aureus* (Cell : Bacteria = 2 : 1). To assess the phagocytosis capacity, RAW264.7 were co-incubated with GFP-*S. aureus* for 4 h. Then cells were washed with PBS to remove the bacteria that had not been phagocytosed. The cytoskeleton and nucleus were labeled with Actin-Tracker Red-Rhodamine (Beyotime, China) and DAPI (Servicebio, China), respectively. Finally, the phagocytosis of bacteria by RAW264.7 was observed using the confocal microscope (ZEISS LSM 710, Germany). To assess the bactericidal capacity, RAW264.7 were lysed using 0.1 % Triton-X100 and mixed with the culture supernatant. The mixed bacterial solution was diluted and spread to blood agar plates. Single colonies were counted after incubation at 37°C overnight. Meanwhile, scanning electron microscope (SEM) examination was further employed and *S. aureus* samples was fixed with 2.5% glutaraldehyde at 4°C for 12 h, then dehydrated using ethanol gradients (30-100%), with each gradient lasting 10 min. After dehydration, the samples were placed in a critical point dryer for approximately 1 h. Finally, the samples were fixed onto the specimen holder using conductive adhesive tape, gold-coated, and prepared for testing using SEM (Hitachi SU8010, Japan).

***Establishment of IAIs relapse models***

To establish implant-associated relapse osteomyelitis models, implant removal and thorough debridement were conducted 11 days after treatment of primary osteomyelitis. Then vancomycin was injected intravenously two times. The procedure simulated the clinical surgical management dealing with IAIs. Subsequent re-insertion of new implants and injection of *S. aureus* into femur was implemented to construct the relapse femur osteomyelitis model.

***Assessment of biosafety***

To assess the biosafety of nanovesicles, the body weight of mice was closely monitored through the whole treatment. The levels of alanine aminotransferase (ALT), aspartate aminotransferase (AST), creatinine (CREA), urea nitrogen (UREA), total protein (TP), and total bilirubin (TBIL) levels in the serum were tested according to the manufacturer’s recommendations. The H&E staining of the major organs (heart, liver, spleen, lung, and kidney) was conducted to assess the pathological changes.


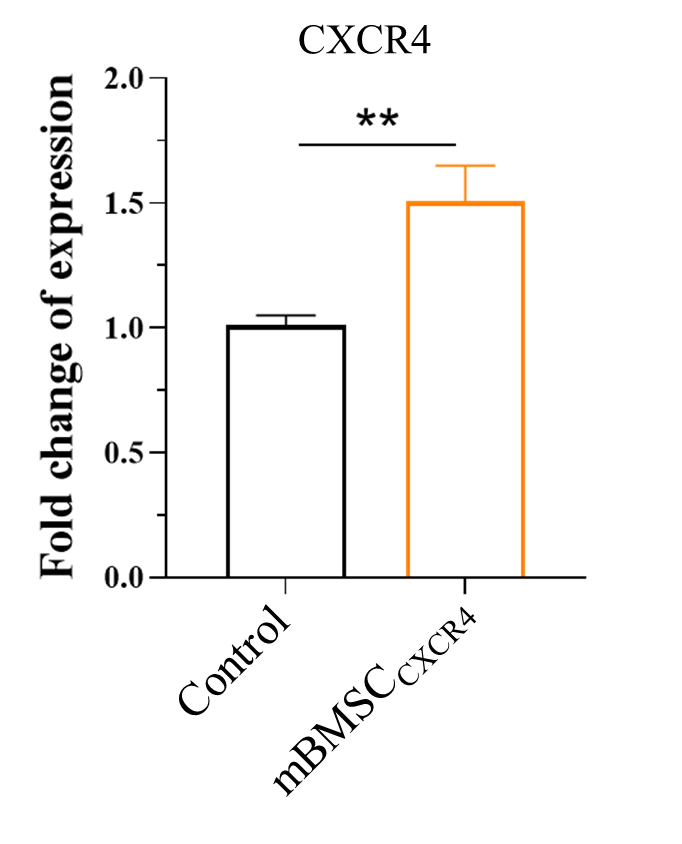


**Figure S1.** Quantitative analysis of CXCR4 expression in BMSCs before and after genetic engineering. Na^+^/K^+^ ATPase serves as a loading control (mean ± SD, n = 3).

**
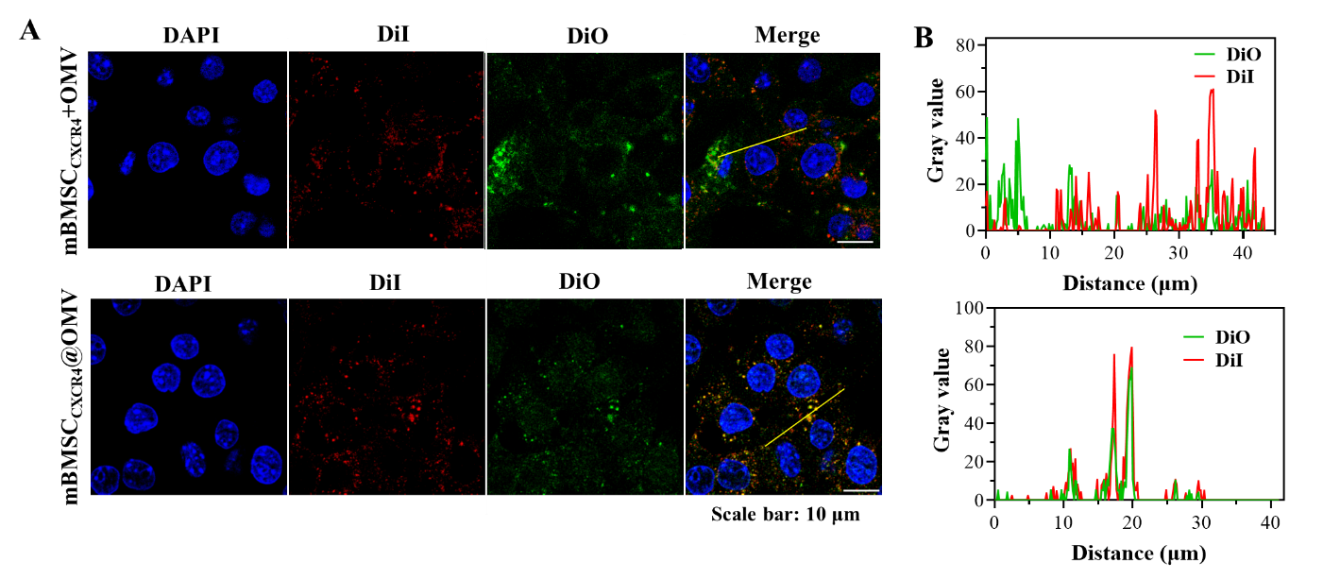
**

**Figure S2.** Co-localization analysis of mBMSC_CXCR4_ and OMV. **(A)** Colocalization analysis by confocal laser scanning microscopy (CLSM) of DiI-labeled mBMSC_CXCR4_ (red fluorescence) and DiO-labeled OMV (green fluorescence). **(B)** The colocalization analysis of fluorescence in mBMSC_CXCR4_ + OMVs (up) and mBMSC_CXCR4_@OMVs (down) by image-J.


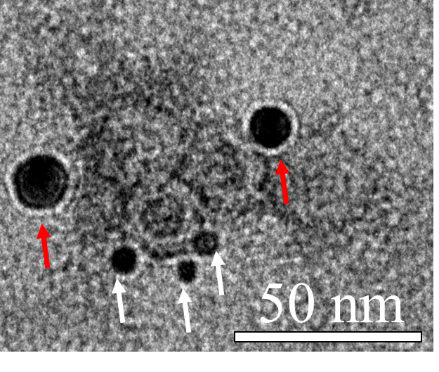


**Figure S3.** Simultaneous binding of both sizes of gold nanoparticles by mBMSC_CXCR4_@OMV. His-tag and CXCR4 antibodies, labeled with 5 nm (white arrow) and 10 nm (red arrow) gold nanoparticles, respectively, were used to identify the hybridization of OMVs with mBMSC_CXCR4_.


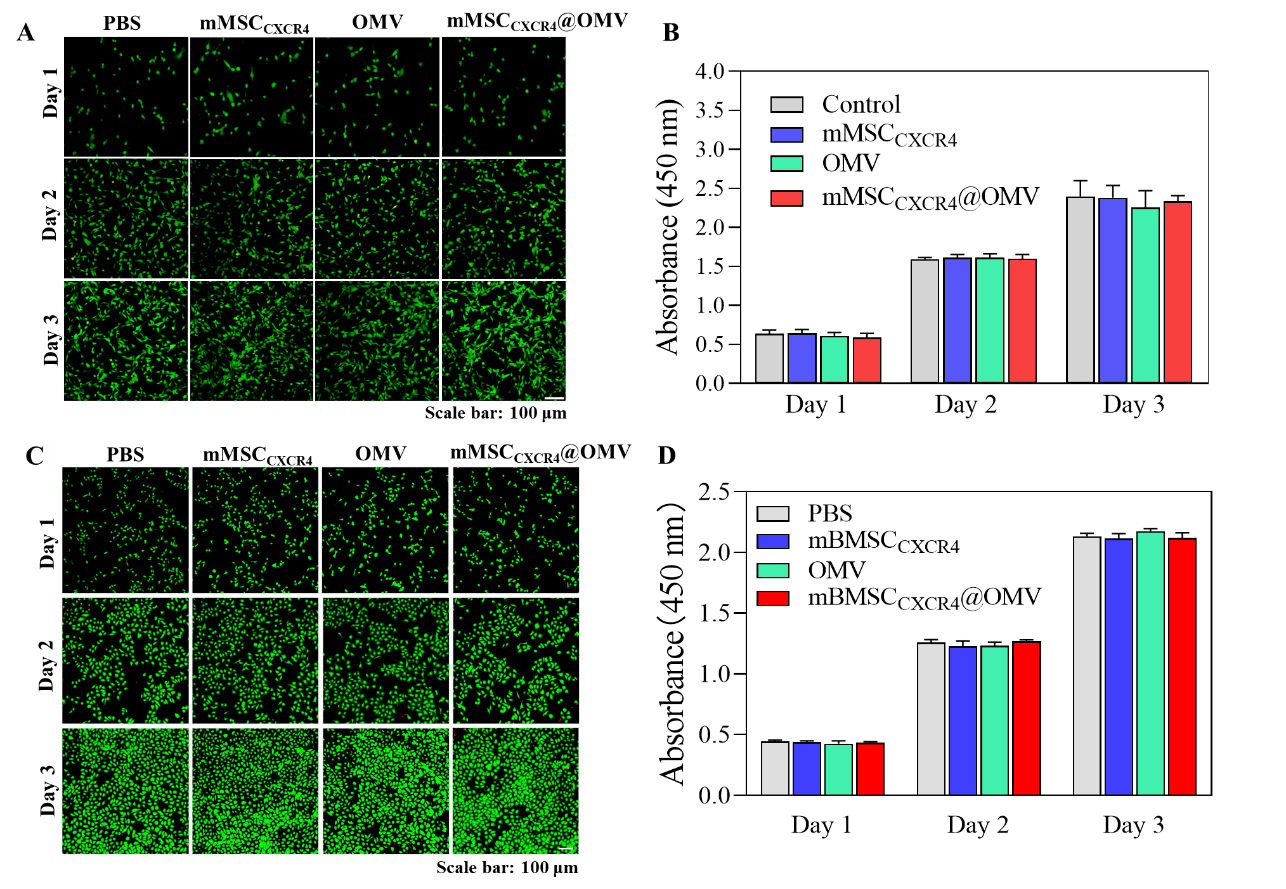


**Figure S4.** The toxicity of mBMSC_CXCR4_@OMV on BMSCs and HUVECs. **(A)** Live and dead staining and **(B)** quantitative analysis of BMSCs following treatment of mBMSC_CXCR4_, OMV, and mBMSC_CXCR4_@OMV for 1, 2, and 3 days. **(C)** Live and dead staining and **(D)** quantitative analysis of HUVECs following treatment of mBMSC_CXCR4_, OMV, and mBMSC_CXCR4_@OMV for 1, 2, and 3 days (mean ± SD, n = 3).


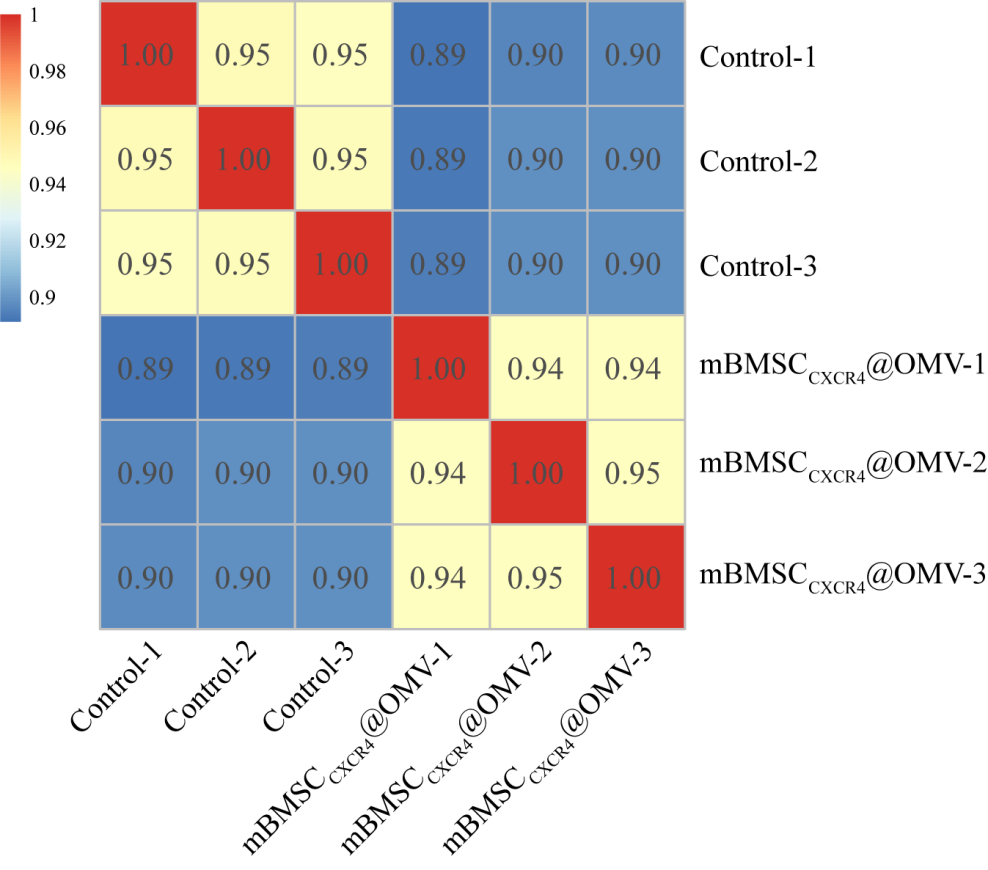


**Figure S5.** The heat map of expression correlation between samples.

**
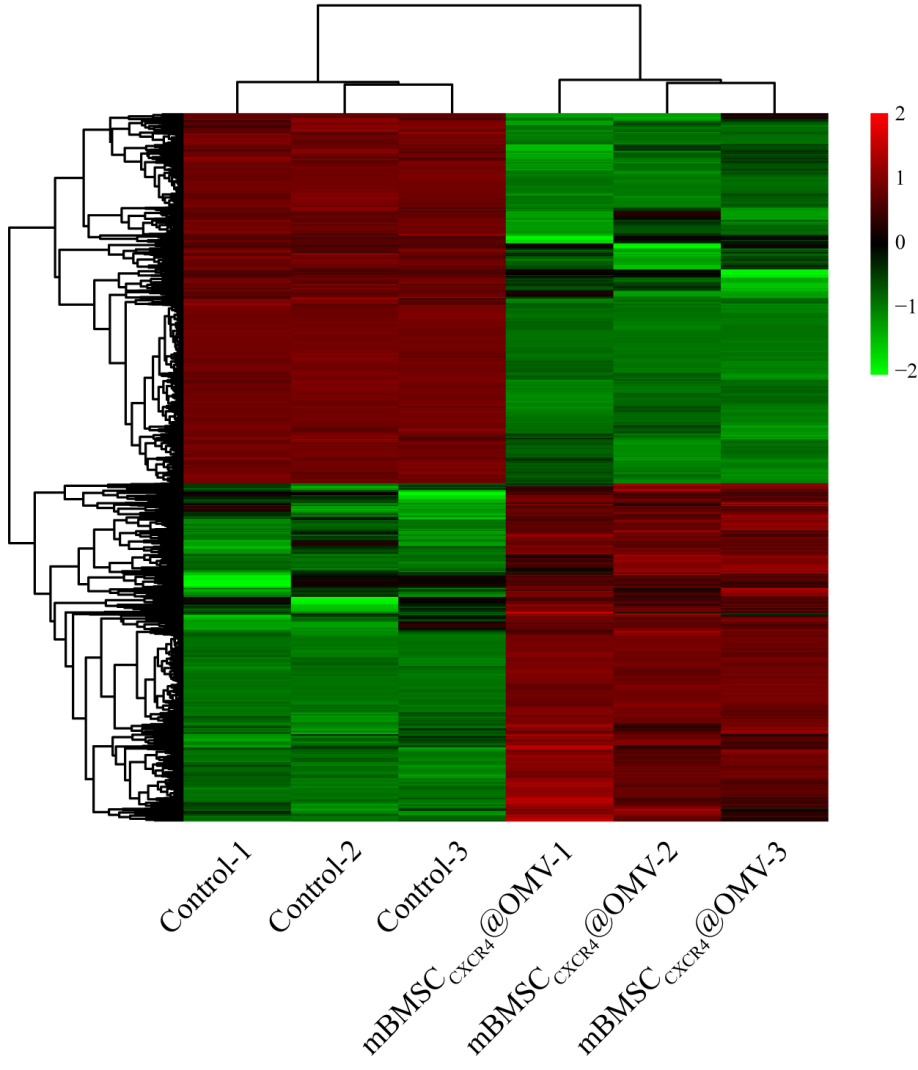
**

**Figure S6.** The heat map of differential expressed genes.


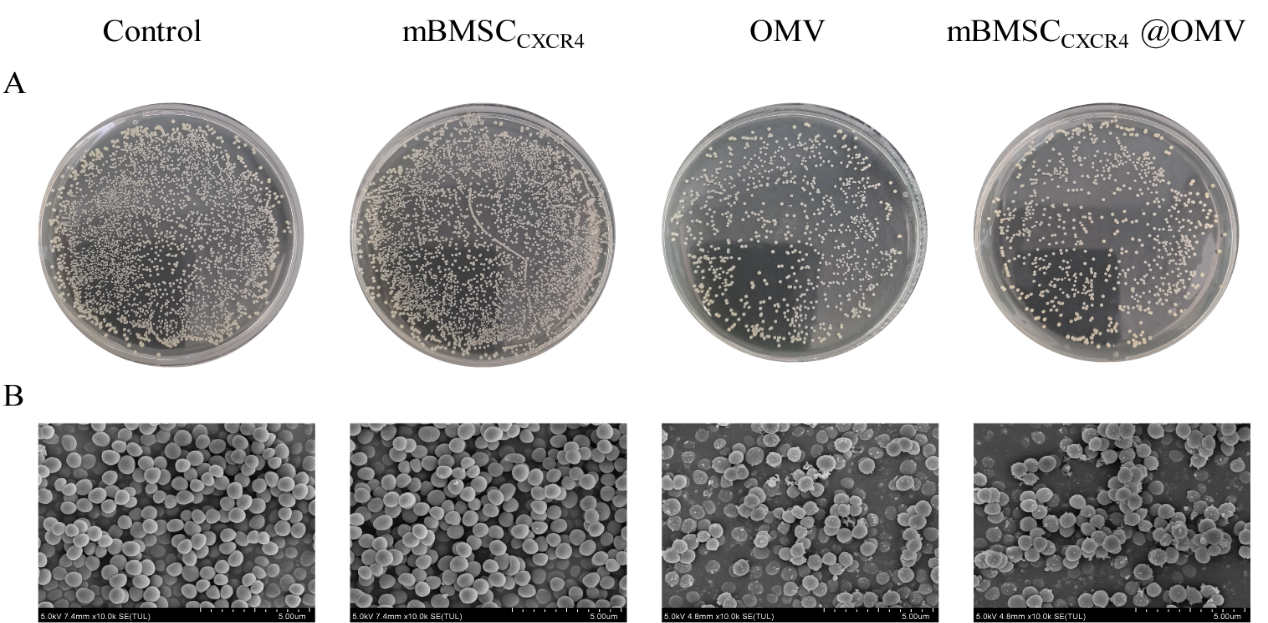


**Figure S7.** The CFU counts **(A)** and SEM images **(B)** of *S. aureus* incubated with RAW264.7 following treatment of PBS, mBMSC_CXCR4_, OMV, or mBMSC_CXCR4_@OMV.


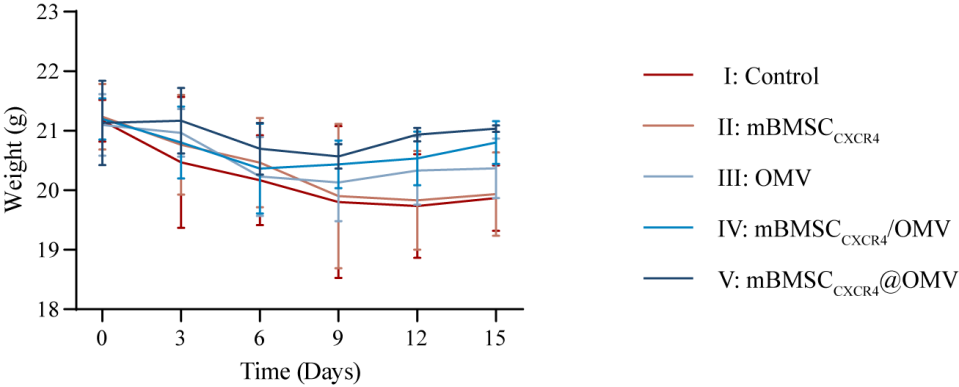


**Figure S8.** The body weight of mice during the whole process of primary IAIs after intravenously injection with saline, mBMSC_CXCR4_, OMV, mBMSC_CXCR4_/OMV and mBMSC_CXCR4_@OMV.


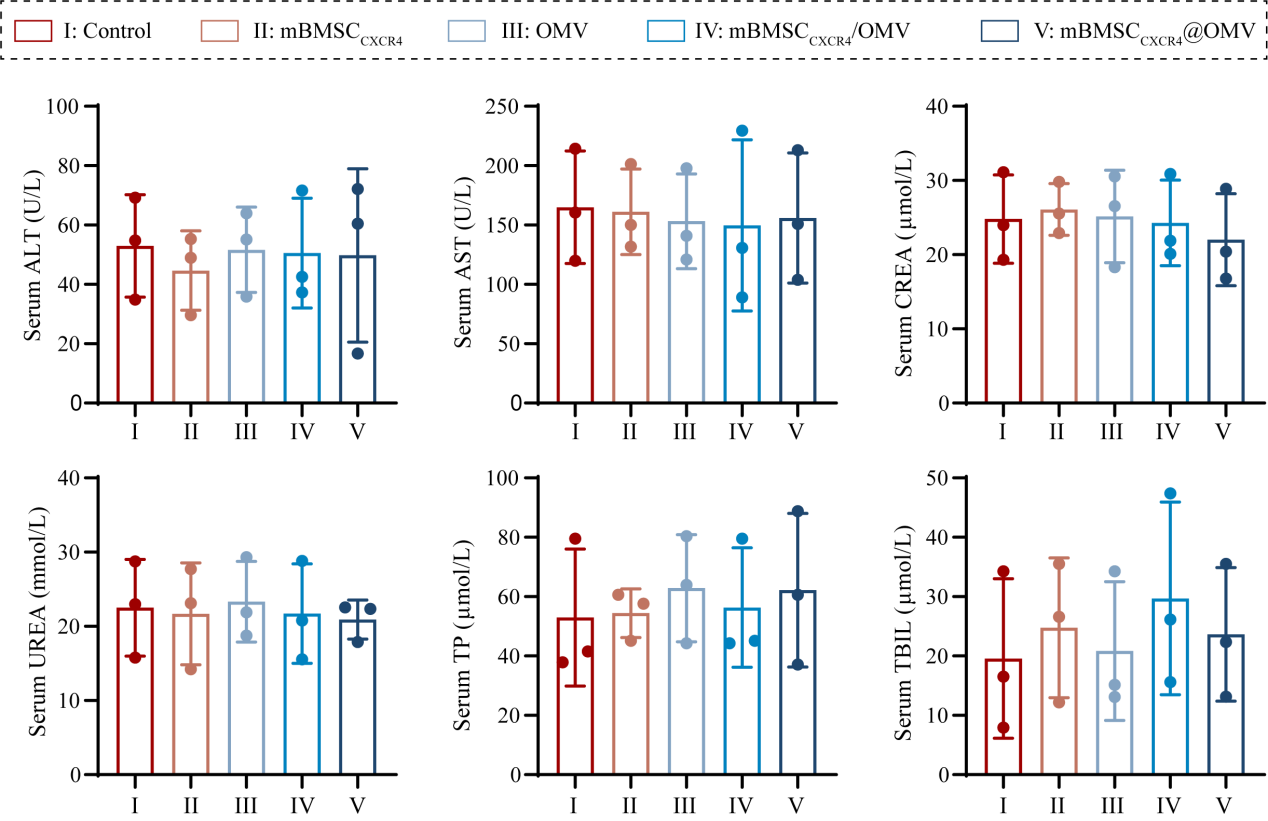


**Figure S9.** Serum biochemical analysis of ALT, AST, CREA, UREA, TP, and TBIL. ALT: Alanine aminotransferase. AST: Aspartate aminotransferase. CREA: Creatine. UREA: Urea nitrogen. TP: Total protein. TBIL: Total bilirubin.


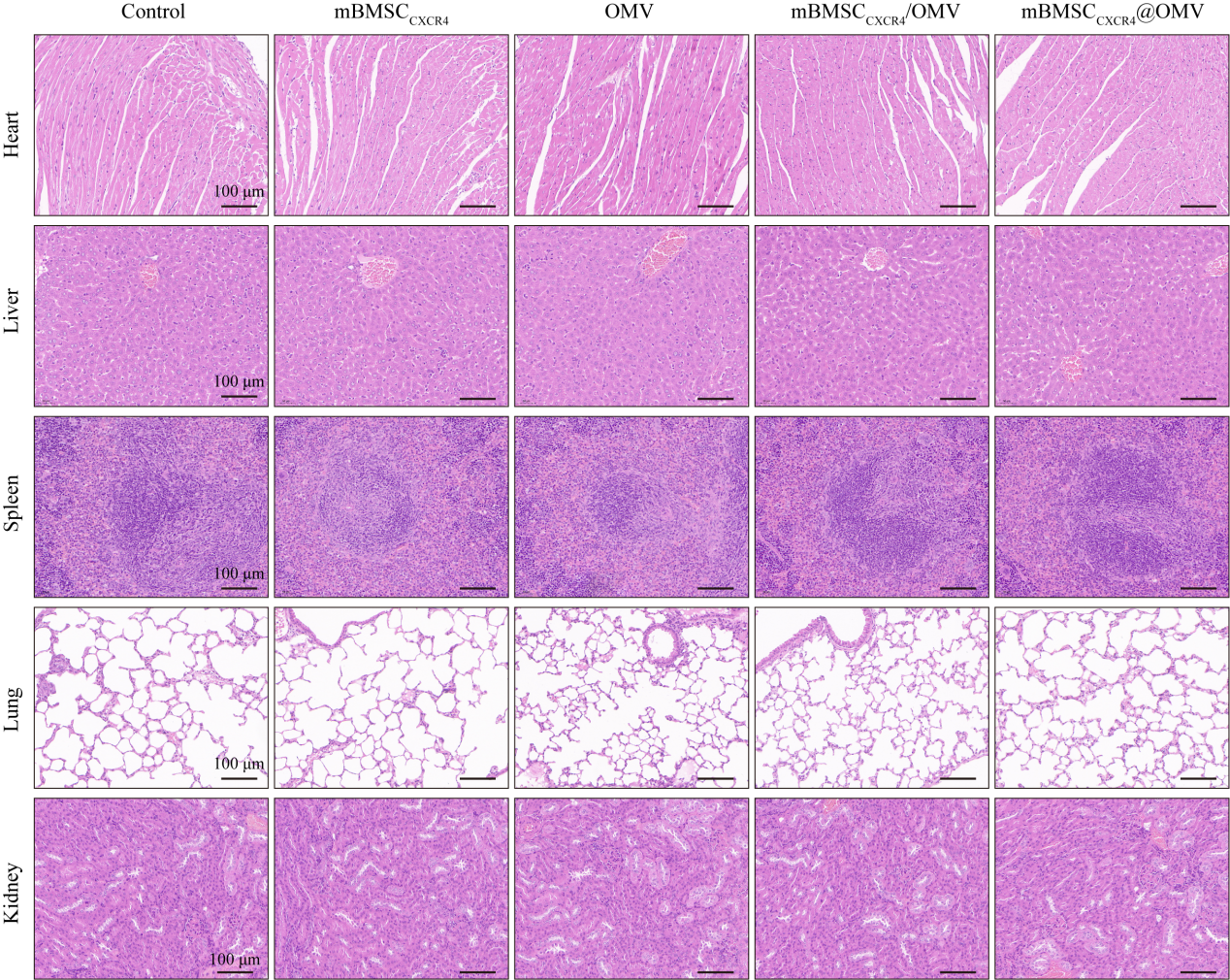


**Figure S10.** The H&E staining images of heart, liver, spleen, lung, and kidney.


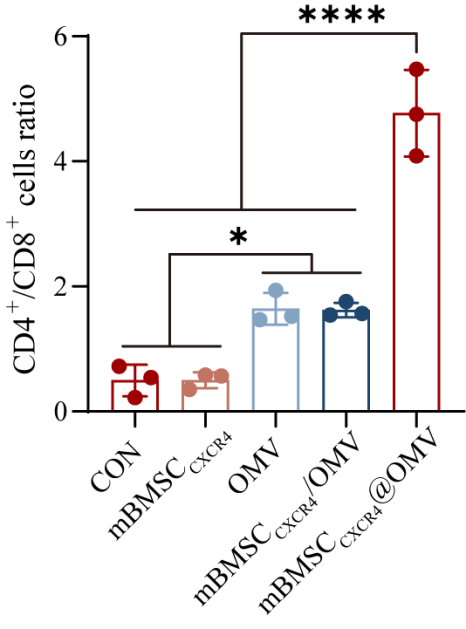


**Figure S11.** The ratio of CD4^+^ T cells to CD8^+^ T cells in bone marrow. (mean ± SD, n = 3).


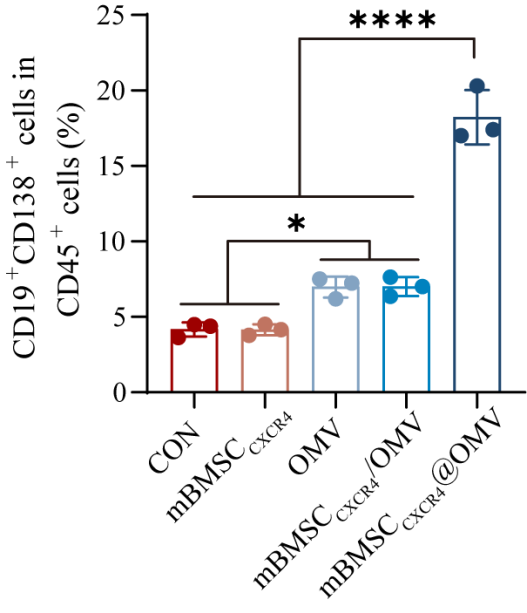


**Figure S12.** Quantification of plasmablasts (CD19^+^CD138^+^) gating on CD45^+^ cells in bone marrow. (mean ± SD, n = 3).


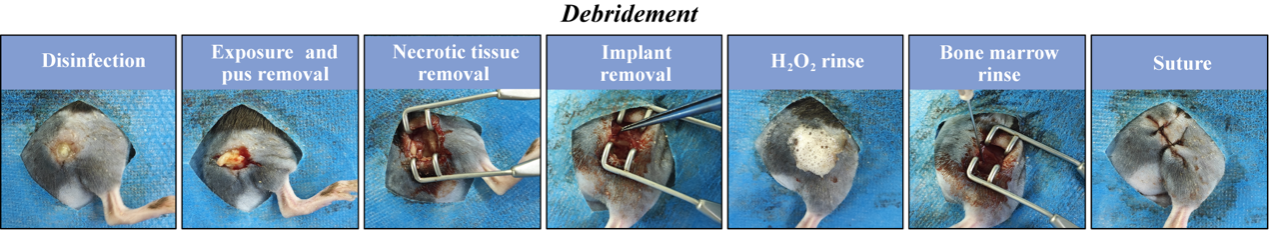


**Figure S13.** The surgical process for debridement.


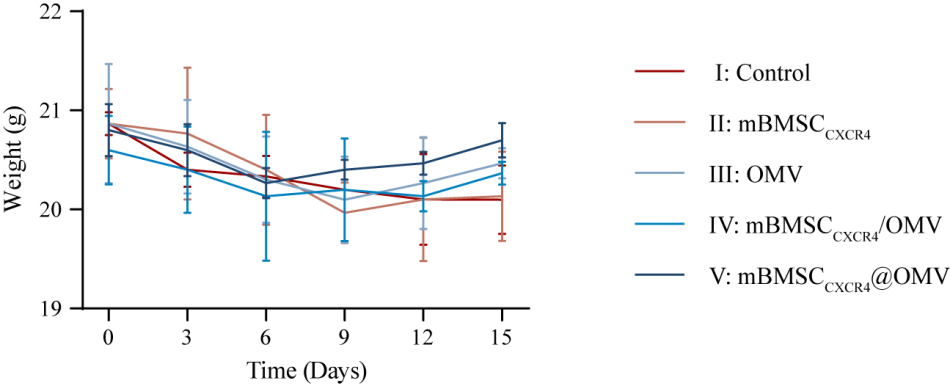


**Figure S14.** The body weight of mice during the whole of recurrent infection after intravenously injection with saline, mBMSC_CXCR4_, OMV, mBMSC_CXCR4_/OMV and mBMSC_CXCR4_@OMV.

**Table S1.** Detailed sequences for constructed plasmids.

| **Amino acid sequence of**  **CXCR4** | MGYQKKLRSMTDKYRLHLSVADLLFVITLPFWAVDAMADWYFGKFLCKAVHIIYTVNLYSSVLILAFISLDRYLAIVHATNSQRPRKLLAEKAVYVGVWIPALLLTIPDFIFADVSQGDISQGDDRYICDRLYPDSLWMVVFQFQHIMVGLVLPGIVILSCYCIIISKLSHSKGHQKRKALKTTVILILAFFACWLPYYVGISIDSFILLGVIKQGCDFESIVHKWISITEALAFFHCCLNPILYAFLGAKFKSSAQHALNSMSRGSSLKILSKGKRGGHSSVSTESESSSFHSS |
| --- | --- |
| **Nucleic acid sequence of CXCR4** | ATGGGCTATCAGAAAAAACTGCGCAGCATGACCGATAAATATCGCCTGCATCTGAGCGTGGCGGATCTGCTGTTTGTGATTACCCTGCCGTTTTGGGCGGTGGATGCGATGGCGGATTGGTATTTTGGCAAATTTCTGTGCAAAGCGGTGCATATTATTTATACCGTGAACCTGTATAGCAGCGTGCTGATTCTGGCGTTTATTAGCCTGGATCGCTATCTGGCGATTGTGCATGCGACCAACAGCCAGCGCCCGCGCAAACTGCTGGCGGAAAAAGCGGTGTATGTGGGCGTGTGGATTCCGGCGCTGCTGCTGACCATTCCGGATTTTATTTTTGCGGATGTGAGCCAGGGCGATATTAGCCAGGGCGATGATCGCTATATTTGCGATCGCCTGTATCCGGATAGCCTGTGGATGGTGGTGTTTCAGTTTCAGCATATTATGGTGGGCCTGGTGCTGCCGGGCATTGTGATTCTGAGCTGCTATTGCATTATTATTAGCAAACTGAGCCATAGCAAAGGCCATCAGAAACGCAAAGCGCTGAAAACCACCGTGATTCTGATTCTGGCGTTTTTTGCGTGCTGGCTGCCGTATTATGTGGGCATTAGCATTGATAGCTTTATTCTGCTGGGCGTGATTAAACAGGGCTGCGATTTTGAAAGCATTGTGCATAAATGGATTAGCATTACCGAAGCGCTGGCGTTTTTTCATTGCTGCCTGAACCCGATTCTGTATGCGTTTCTGGGCGCGAAATTTAAAAGCAGCGCGCAGCATGCGCTGAACAGCATGAGCCGCGGCAGCAGCCTGAAAATTCTGAGCAAAGGCAAACGCGGCGGCCATAGCAGCGTGAGCACCGAAAGCGAAAGCAGCAGCTTTCATA GCAGC |

**Table S2**. Flow antibodies used to detect the uptake of mBMSC_CXCR4_@OMV by immune cells in bone marrow.

|  | **Antibody** | **Color** | **Cat.** | **Company** | **Country** |
| --- | --- | --- | --- | --- | --- |
| **Macrophages** | CD45 | FITC | 553079 | BD Pharmingen | USA |
|  | CD11b | PerCP-Cy5.5 | 550993 | BD Pharmingen | USA |
| **Neutrophils** | CD45 | FITC | 553079 | BD Pharmingen | USA |
|  | CD11b | PerCP-Cy5.5 | 550993 | BD Pharmingen | USA |
|  | Ly-6G | PE | 551461 | BD Pharmingen | USA |
| **DCs** | CD45 | FITC | 553079 | BD Pharmingen | USA |
|  | CD11c | PE | 557401 | BD Pharmingen | USA |
| **T cells** | CD45 | FITC | 553079 | BD Pharmingen | USA |
|  | CD3 | PE-Cy7 | 552774 | BD Pharmingen | USA |
| **B cells** | CD45 | FITC | 553079 | BD Pharmingen | USA |
|  | CD19 | PE-Cy7 | 552854 | BD Pharmingen | USA |

**Table S3**. Flow antibodies used *in vitro*.

|  | **Antibody** | **Color** | **Cat.** | **Company** | **Country** |
| --- | --- | --- | --- | --- | --- |
| **Macrophages** | F4/80 | PE | 565410 | BD Pharmingen | USA |
|  | CD86 | PE-Cy7 | 560582 | BD Pharmingen | USA |
|  | MHC-II | FITC | 562009 | BD Pharmingen | USA |

**Table S4**. Flow antibodies used to detect immune cells in bone marrow.

|  | **Antibody** | **Color** | **Cat.** | **Company** | **Country** |
| --- | --- | --- | --- | --- | --- |
| **Macrophages** | CD45 | FITC | 553079 | BD Pharmingen | USA |
|  | CD11b | PerCP-Cy5.5 | 550993 | BD Pharmingen | USA |
|  | F4/80 | PE | 565410 | BD Pharmingen | USA |
|  | CD86 | PE-Cy7 | 560582 | BD Pharmingen | USA |
|  | CD206 | APC | 568808 | BD Pharmingen | USA |
| **DCs** | CD45 | FITC | 553079 | BD Pharmingen | USA |
|  | CD11C | PE | 557401 | BD Pharmingen | USA |
|  | CD80 | APC | 560016 | BD Pharmingen | USA |
|  | CD86 | PE-Cy7 | 560582 | BD Pharmingen | USA |
| **T cells** | CD45 | FITC | 553079 | BD Pharmingen | USA |
|  | CD3 | APC | 553066 | BD Pharmingen | USA |
|  | CD4 | PE | 557308 | BD Pharmingen | USA |
|  | CD4 | PerCP-Cy5.5 | 551162 | BD Pharmingen | USA |
| **B cells** | CD45 | FITC | 553079 | BD Pharmingen | USA |
|  | CD19 | PE-Cy7 | 552854 | BD Pharmingen | USA |
|  | CD138 | APC | 558626 | BD Pharmingen | USA |
| **MDSC** | CD45 | FITC | 553079 | BD Pharmingen | USA |
|  | CD11b | PerCP-Cy5.5 | 550993 | BD Pharmingen | USA |
|  | Gr-1 | APC | 553129 | BD Pharmingen | USA |
| **Memory B cells** | CD45 | FITC | 553079 | BD Pharmingen | USA |
|  | CD19 | PE-Cy7 | 552854 | BD Pharmingen | USA |
|  | IgG | PE | 405307 | Biolegend | USA |
|  | IgD | APC | 560868 | BD Pharmingen | USA |
